# Phytochemistry and Anti-Inflammatory and Antioxidant Activities of *Cinnamomum osmophloeum* and Its Bioactive Constituents: A Review

**DOI:** 10.3390/plants14040562

**Published:** 2025-02-12

**Authors:** Renan Oliveira Silva Damasceno, João Lucas Silva Pinheiro, Lorena Duarte da Silva, Lucas Henrique Marques Rodrigues, Jeremias Justo Emídio, Tamires Cardoso Lima, Damião Pergentino de Sousa

**Affiliations:** 1 Department of Physiology and Pharmacology, Federal University of Pernambuco, Recife 50670-901, Pernambuco, Brazil; jaolucas.1424@gmail.com (J.L.S.P.); lorenaduarte.silva@ufpe.br (L.D.d.S.); lucas.henriquemarques@ufpe.br (L.H.M.R.); 2 Department of Pharmaceutical Sciences, Federal University of Paraíba, João Pessoa 58051-970, Paraíba, Brazil; jeremiasjusto@gmail.com; 3 Department of Pharmacy, Federal University of Sergipe, São Cristóvão 49100-000, Sergipe, Brazil; tamires.cl87@gmail.com

**Keywords:** *Cinnamomum osmophloeum*, essential oils, phytochemistry, anti-inflammatory, antioxidant, natural products, medicinal plant, drugs, oxidative stress, pharmacological activity

## Abstract

*Cinnamomum osmophloeum*, commonly known as indigenous cinnamon, is a tree species native to Taiwan’s hardwood forests. It has been extensively investigated for its chemical composition and bioactivities. Several reports have shown that *C. osmophloeum* leaves are rich in aromatic oils, which are grouped into various chemotypes based on their major constituents. Components of the volatile oils included phenylpropanoids, monoterpenoids, sesquiterpenoids, phenols, coumarins, and other miscellaneous compounds. In addition, other secondary metabolites previously identified in this species included flavonol glycosides, phenolic acids, lignans, proanthocyanidins, and cyclopropanoids. *C. osmophloeum* is widely recognized for its medicinal and industrial applications, particularly its essential oils. In general, essential oils exhibit remarkable anti-inflammatory and antioxidant actions, enabling them to modulate key inflammatory mediators and neutralize free radicals. This review explored the phytochemical composition of the essential oils and extracts from *C. osmophloeum* as well as therapeutic potential of this species, focusing on the action mechanisms and clinical potential. We hope that this review will contribute to a better understanding of the biological effects of this plant and its potential applications in the management of conditions associated with inflammation and oxidative stress.

## 1. Introduction

**Figure 1:**
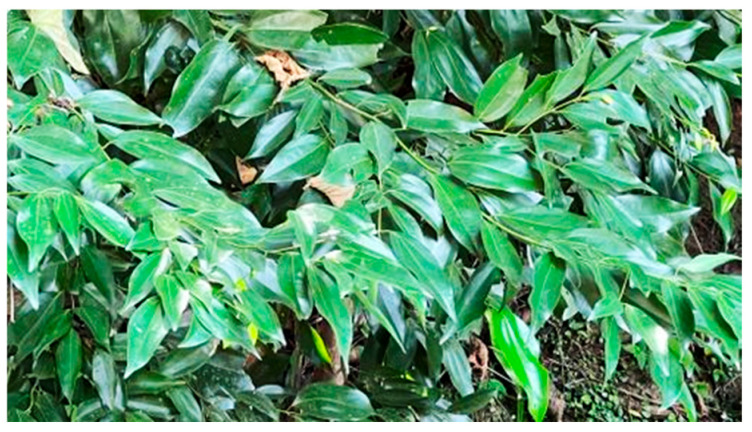
Species *Cinnamomum osmophloeum* Kanehira. Licensed under http://creativecommons.org/licenses/by-nc/4.0/ (accessed on 20 January 2025).

The *Cinnamomum osmophloeum* Kanehira species is a medium-sized evergreen tree that belongs to the genus *Cinnamomum* (Lauraceae family) and is commonly known as indigenous cinnamon or pseudo-cinnamon (Figure 1). This plant grows primarily in the subtropical biome, being endemic to the natural hardwood forests of Central and Northern Taiwan [1,2,3]. Pseudo-cinnamon is an important herb in traditional Chinese medicine, with a wide range of applications in the culinary context, cosmetics, and folk medicine, and as a flavoring agent. This medicine possesses commercial value and is of great interest to researchers because of its bioactive constituents and similar chemical composition to *Cinnamomum cassia* oil, commonly used in food and beverages [4].

Several chemical studies have investigated the phytochemistry of *C. osmophloeum*, including the chemical composition of the essential oils and crude extracts, which will be discussed later in this review. Many reports have shown that indigenous cinnamon leaves and twigs are rich in essential oils, and various chemotypes with different secondary metabolite profiles were identified in the leaves. In addition, other secondary metabolites also identified in this species included flavonoid aglycones and glycosides, phenolic acids, lignans, phenylpropanoids, coumarins, proanthocyanidins, and cyclopropanoids [5,6].

The effects of the essential oils from *C. osmophloeum* on inflammatory response and oxidative stress have been well documented. Studies have shown significant anti-inflammatory effects by reducing inflammatory mediators, such as pro-inflammatory cytokines and nitric oxide (NO), which are elevated in response to endotoxins. These effects are mediated by inhibiting key proteins involved in the inflammatory response, such as the nuclear factor kappa B (NF-κB) [7]. Furthermore, they promote the neutralization of free radicals, mitigating oxidative damage to lipids, proteins, and nucleic acids [8]. These biological effects have been attributed to key compounds such as cinnamaldehyde and linalool, highlighting essential oils as promising alternatives for developing natural biological modulators.

In the last review on this species, the authors commented on dietary, phytochemical, and pharmacological aspects [6]. In this review, the data were organized focusing on the mechanisms of action of the anti-inflammatory and antioxidant activities of *C. osmophloeum* and its chemical components, with the information being discussed with illustrations for a better understanding of the pharmacotherapeutic potential of the medicinal plant. By providing a comprehensive overview of the current scientific evidence, we hope to establish a strong foundation for the future clinical applications of these bioactive compounds, thereby contributing to the expanded use of *C. osmophloeum* in therapeutic settings.

## 2. Phytochemistry Characterization of *C. osmophloeum*

### 2.1. Essential Oils Composition of C. osmophloeum

The leaves and twigs from *C. osmophloeum* are rich in aromatic oils. Major components of the volatile oils from *C. osmophloeum* have been analyzed by Gas Chromatography–Mass Spectrometry (GC/MS) and different secondary metabolites have been identified, including monoterpenoids, sesquiterpenoids, alcohols, phenols, aldehydes, ketones, esters, coumarins, acids, and other miscellaneous compounds [5].

Chemotypes are chemically distinct entities in the same plant species, varying the composition of their secondary metabolites. The plants can create different chemotypes distinguished based on the dominant compounds in the essential oil. The chemical composition of a plant is primarily determined by its genetic characteristics; however, other factors like nutrients, the composition of the soil, climatic and environmental conditions, and altitude can influence the quantitative and qualitative composition of the essential oils. Thus, knowing the chemotype of an essential oil is very important since the presence of different chemotypes can alter the bioactivity of the oil [9].

**Figure 2:**
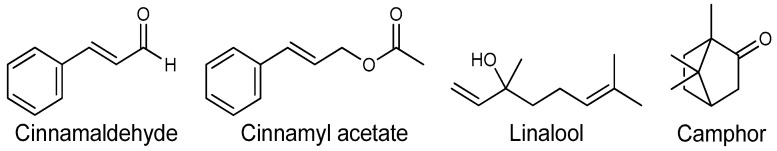
Chemical structures of the main volatile components found in *C. osmophloeum* chemotypes.

According to some authors, *C. osmophloeum* leaf essential oils can be classified into nine chemotypes according to the dominant compounds, namely, the cassia type, cinnamaldehyde type, coumarin type, linalool type, eugenol type, camphor type, terpineol-4-ol type, linalool-terpineol type, and mixed type [10]. Already, other authors have reported that the *C. osmophloeum* leaf essential oil comprises only six chemotypes: cinnamaldehyde, cinnamaldehyde/cinnamyl acetate, cinnamyl acetate, linalool, camphor, and mixed types [5,11]. Herein, we adopt the classification into six chemotypes. Figure 2 contains the chemical structures of cinnamaldehyde, cinnamyl acetate, linalool, and camphor, the main volatile components found in *C. osmophloeum* chemotypes.

Lin and colleagues (2007) investigated the chemotypes of 92 leaf essential oils of *C. osmophloeum* from a clonal orchard at the Chinese Culture University’s Hwa-Lin Experimental Forest in Taipei County, with cuttings of trees from 13 natural populations from Central, Eastern, and Southern regions of Taiwan. Among the investigated specimens, the main chemotypes classified in the individual cutting clones were the cinnamaldehyde type (50 plants, representing 50–95% of the total volatiles), linalool type (1 plant, 73.9%), β-cubebene type (2 plants, 59.4%, and 78.7%), cinnamyl acetate type (1 plant, 61.8%), and mixed type (38 plants, ≥20% of each compound) [8].

The cinnamaldehyde chemotype is the major type identified in *C. osmophloeum*. Most prior studies have shown that the most abundant chemical component contents of the cinnamaldehyde chemotype were cinammaldehyde/*trans*-cinnamaldehyde [1,2,11,12,13,14,15,16,17,18,19,20,21,22,23,24,25,26], benzaldehyde [12,13,15,17,19,20,23,25,26], benzenepropanal [11,15,17,18,19,23,24], and cinnamyl acetate/*trans*-cinnamyl acetate [1,2,15,16,21,24,26]. Furthermore, there was a complementary relationship between the content of *trans*-cinnamaldehyde and *trans*-cinnamyl acetate in leaf oils and their seasonal variations in *C. osmophloeum* [27].

The cinnamaldehyde/cinnamyl acetate chemotype encompassed as principal components cinnamyl acetate, *trans*-cinnamaldehyde, and benzenepropanal [11,14,15,16,18]. The cinnamyl acetate type comprised cinnamyl acetate [1,11,14,16,18], 2-methylbenzofuran [11,14,16,18], and geranyl acetate [11,14,16,18]. The linalool chemotype contained linalool [11,14,15,16,18,28,29,30] and cinnamaldehyde/*trans*-cinnamaldehyde [14,16,18,28,29,30] as major components. The camphor type contained camphor/D-(+)-camphor, L-bornyl acetate [1,2,11,14,15,16,18], and limonene [14,15,16,18]. Finally, the mixed type included spathulenol [14,18,31], neral [12,13,18,20], L-bornyl acetate/bornyl acetate [1,4,11,15,16,32], 1,8-cineole [12,20,31], geranial [18], and τ-cadinol and α-cadinol [1,11,15,16,32]. Table 1 exhibits the chemotypes, volatile compounds, and their relative contents (%) of the essential oils from *C. osmophloeum*.

### 2.2. Phytoconstituents of the Extracts from C. osmophloeum

**Figure 3:**
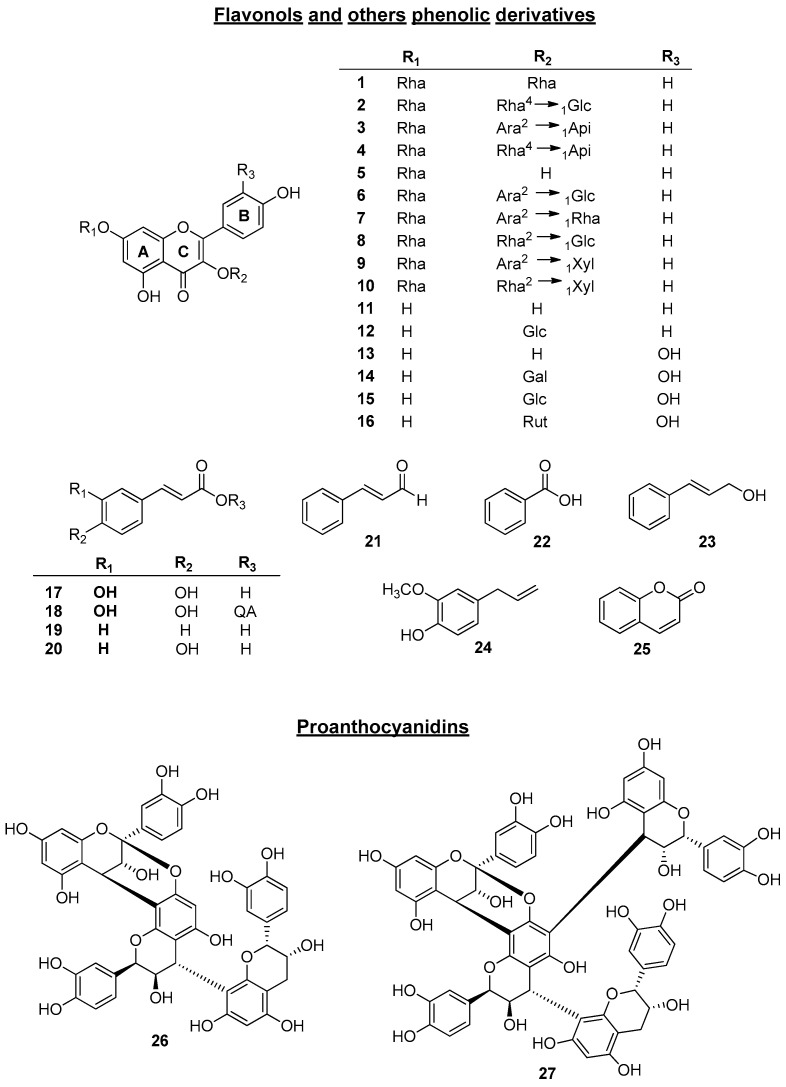
Chemical structures of the flavonols, phenolic derivatives, and proanthocyanidins isolated/identified in *C. osmophloeum*. Abbreviations: Rha, rhamnopyranoside; Glc, glucopyranoside; Ara, arabinofuranoside; Api, apiofuranoside; Xyl, xylopyranoside; Gal, galactopyranoside; Rut, rutinoside; QA, quinic acid. The letters A, B, and C represent the rings of the basic flavonoid structure.

**Figure 4:**
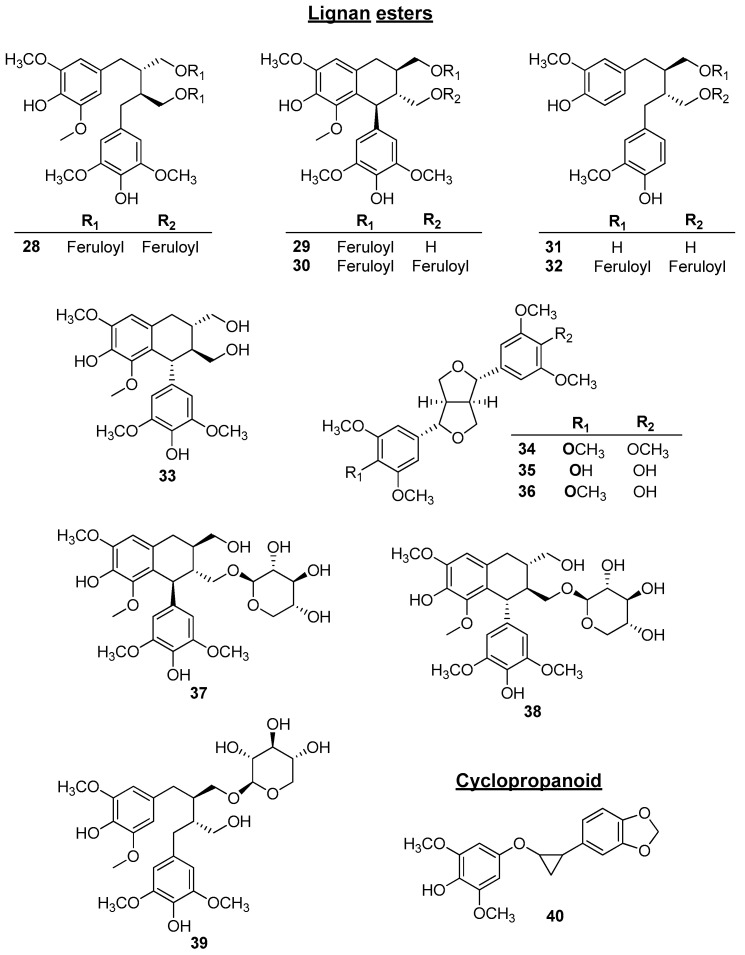
Chemical structures of the lignans and cyclopropanoid isolated/identified in *C. osmophloeum*.

Most of the studies on the chemical composition of *C. osmophloeum* were conducted with essential oils. The extraction and isolation of the phytoconstituents of this species began only in the early 2000s. The first phytochemical study on this species was performed in 2005 by Fang and colleagues [33]. The secondary metabolites previously isolated from *C. osmophloeum* included flavonoids, mainly flavonol glycosides, phenolic acids, lignans, proanthocyanidins, and cyclopropanoids. The chemical structures of all the phytoconstituents isolated from *C. osmophloeum* are shown in Figure 3 and Figure 4. Table 2 summarizes the reported non-essential oil metabolites of *C. osmophloeum,* the plant material, the type of extract, and the method used for characterization/identification of these metabolites.

Flavonol glycosides are the most common and representative phytoconstituents of *C. osmophloeum* in addition to the essential oil constituents. Sixteen flavonols (**1**–**16**) were previously identified in *C. osmophloeum*. Fang and colleagues [33] isolated and elucidated four flavonol glycosides from the methanol extract of *C. osmophloeum* leaves. The flavonol kaempferol 3,7-*O*-dirhamnoside (**1**, kaempferitrin, 3.4 g) was obtained from the chloroform fraction, whereas the kaempferol 3-*O*-β-D-glucopyranosyl-(1→4)-α-L-rhamnopyranosyl-7-*O*-α-L-rhamnopyranoside (**2**, 320 mg), kaempferol 3-*O*-β-D-apiofuranosyl-(1→2)-α-L-arabinofuranosyl-7-*O*-α-L-rhamnopyranoside (**3**, 15 mg), and kaempferol 3-*O*-β-D-apiofuranosyl-(1→4)-α-L-rhamnopyranosyl-7-O-α-L-rhamnopyranoside (**4**, 8 mg) were isolated from the *n*-butanol fraction [33]. The kaempferol glycosides **2** and **3** were later isolated from the methanol extract *C. osmophloeum* (*n*-butanol fraction) by Lee and colleagues [34] using the same extraction and isolation procedures described previously [33]. Lin and colleagues (2011) investigated the composition of the water extract from *C. osmophloeum* leaves by High-Performance Liquid Chromatography (HPLC) and identified and characterized as the main compounds two compounds previously isolated [33], the flavonols **1** and **2** [35].

In 2009, another flavonol, kaempferol-7-*O*-rhamnoside (**5**) was extracted from ethanolic extract (butanol fraction) from the twigs of *C. osmophloeum*, purified by semipreparative HPLC and elucidated by 1D and 2D Nuclear Magnetic Resonance (NMR) data and Mass Spectrometry [36]. In 2013, Wu and colleagues evaluated the chemical composition of leaf water extracts from *C. osmophloeum* originating from 11 provenances. The water extract was chromatographically fractionated by preparative HPLC based on the bioassay-guided fractionation procedure, yielding flavonols **1** and **5** [37].

In 2012, nine flavonol glycosides were isolated and identified from the ethanolic extract of twigs from *C. osmophloeum* (*n*-butanol fraction). The compounds were characterized by 1D and 2D NMR and Mass Spectrometry data. The flavonols identified were **1** (152 mg), **2** (22 mg), **3** (1082 mg)**, 5** (33 mg), kaempferol 3-*O*-β-D-glucopyranosyl-(1→2)-α-L-arabinofuranosyl-7-*O*-α-L-rhamnopyranoside (**6**, 241 mg), kaempferol 3-*O*-α-L-rhamnopyranosyl-(1→2)-α-L-arabinofuranosyl-7-*O-*α-L-rhamnopyranoside (**7**, 132 mg), kaempferol 3-*O*-β-D-glucopyranosyl-(1→2)-α-L-rhamnopyranosyl-7-*O*-α-L-rhamnopyranoside (**8**, 21 mg), kaempferol 3-*O*-β-D-xylopyranosyl-(1→2)-α-L-arabinofuranosyl-7-*O-*α-L-rhamnopyranoside (**9**, 25 mg), and kaempferol 3-*O*-β-D-xylopyranosyl-(1→2)-a-L-rhamnopyranosyl-7-*O*-α-L-rhamnopyranoside (**10**, 21 mg). The flavonols **9** and **10** are novel compounds in the literature, whereas **6**, **7**, and **8** were isolated *from C. osmophloeum* for the first time [38].

*C. osmophloeum* leaf extract and its nanoemulsion and hydrosol (a distilled product from cinnamon leaves) were analyzed by Ultra-High-Performance Liquid Chromatography–Tandem Mass Spectrometry (UPLC-MS/MS) to identify and quantity the bioactive compounds. Various phenolic compounds were identified, including kaempferol (**11**), kaempferol 3-β-D-glucopyranoside (**12**, astragalin), quercetin (**13**), quercetin-3-*O*-galactoside (**14**, hyperoside), quercetin-3-*O*-glucoside (**15**, isoquercitrin), quercetin-3-*O*-rutinoside (**16**, rutin), caffeic acid (**17**), 5-*O*-caffeoylquinic acid (**18**, chlorogenic acid), *trans*-cinnamic acid (**19**), *p*-coumaric acid (**20**), cinnamaldehyde (**21**), benzoic acid (**22**), cinnamyl alcohol (**23**), eugenol (**24**), and coumarin (**25**). All the compounds were identified by comparing their retention times and mass spectral data with those of standards and data reported in the literature [39]. Still, in 2023, Wang and colleagues also evaluated the bioactive compounds in *C. osmophloeum* (cinnamon leaves) from ethanol extract, nanoemulsion, and hydrosol by UPLC-MS/MS. These authors found a similar composition to the one related by Huang and Chen (2023), including the compounds **11**–**24** [40].

Two proanthocyanidins, obtained from a proanthocyanidin-rich butanol soluble fraction, were purified by Sephadex LH-20 gel chromatography and identified by Mass Spectrometry. The isolated proanthocyanidins were characterized as epicatechin-(2β→O7,4β→8)-epicatechin-(4β→8)-epicatechin (**26**, cinnamtannin B1) and epicatechin-(2β→O7,4β→8)-[epicatechin-(4β→6)]-epicatechin-(4β→8)-epicatechin (**27**, parameritannin A1) [41].

Twelve lignan esters (**28**–**39**) were obtained from the ethyl acetate and butanol fractions of the roots of *C. osmophloeum* by column chromatography on silica gel and preparative HPLC. Their structures were identified using 1D and 2D NMR Spectroscopy and Mass Spectrometry. The dibenzylbutane-type lignan esters 9,9′-Di-*O*-feruloyl-5,5′-dimethoxysecoisolariciresinol [**28**, (+)-secolyoniresinol diferulate, 80 mg], (7′S,8′R,8R)-lyoniresinol-9-*O*-(*E*)-feruloyl ester [**29**, (+)-lyoniresinol monoferulate, 13 mg], and (7′S,8′R,8R)-lyoniresinol-9,9′-di-*O-*(*E*)-feruloyl ester [**30**, (+)-lyoniresinol monoferulate, 28 mg] were reported for the first time in literature. Among the known lignans, the authors extracted other three dibenzylbutane-type lignans [secoisolariciresinol (**31**, 2 mg), secoisolariciresinol diferulate (**32**, 10.0 mg), and (-)-lyoniresinol (**33**, 17.0 mg)], three tetrahydrofuran-type lignans [(-)-yangambin (**34**, 67.5 mg), (+)-syringaresinol (**35**, 49.8 mg), and (±)-de-4′-*O*-methyl yangambin (**36**, 22.0 mg)], and three lignan xylosides [lyoniside (**37**, 27.0 mg), nudiposide (**38**, 5.8 mg), and ssioriside (**39**, 7.0 mg)] [42].

Finally, Chen and colleagues (2021) reported the isolation and characterization of a new cyclopropanoid, namely, 4-(2-(benzo[d][1,3]-dioxol-5-yl)-cyclopropoxy)-2,6-dimethoxyphenol (**40**, 6 mg), from the stems of *C. osmophloeum*. This compound was obtained from chloroform fraction [43].

## 3. Biological Activities of *C. osmophloeum*

### 3.1. Crude Extracts

Rao and colleagues (2007) investigated the biological activities of three extracts from *C. osmophloeum*—hexane, ethyl acetate, and methanol—evaluating its anti-inflammatory and tumor cell growth inhibitory effects. The results showed that all the tested extracts inhibited the production of the pro-inflammatory mediator NO by reducing inducible nitric oxide synthase (iNOS) expression, as well as levels of tumor necrosis factor (TNF-α) and interleukin-12 (IL-12) in lipopolysaccharide (LPS)/interferon-gamma (IFN-γ) activated murine peritoneal macrophages [44]. Similar results were posteriorly demonstrated with ethanol extracts from *Cinnamomum verum*, which suppressed NO production, NF-κB activation, and the production of TNF-α, interleukin-1β (IL-1β), and interleukin-6 (IL-6) in LPS-stimulated cells [45].

Moreover, Wu and colleagues (2013) [37] examined the antioxidant activities of water extracts from *C. osmophloeum* and their phytochemicals. The authors revealed that the extracts had significant antioxidant activity due to their bioactive compounds, including flavonoid glycosides, such as compounds **1** and **5**. Consistent with this, the literature has already shown that *Cinnamomum* species are rich in several flavonoid glycosides, mainly kaempferol types, with a wide range of health-promoting benefits, such as cardioprotection, neuroprotection, hepatoprotection, and anti-diabetic effects [46,47]. The data also showed that an extract had the highest total phenolic content and better scavenging activity against 2,2-Diphenyl-1-picrylhydrazyl (DPPH) and superoxide radicals, metal chelation, and reducing power [37].

Additionally, an extract from *C. osmophloeum* was tested to explore the correlation between total phenolic content and antioxidant activities. Ho and colleagues (2019) reported that the major bioactive components were *trans*-cinnamaldehyde (87.7%), benzaldehyde (7.0%), and cinnamyl acetate (5.3%), which were responsible for scavenging DPPH radical, metal chelation, and reducing power. The antioxidant ability in binding to the 2,2′-azino-bis(3-ethylbenzothiazoline-6-sulfonic acid (ABTS) radical was slightly less than that of Trolox, and they concluded that the antioxidant activity of the extracts was intrinsically linked to the phenolic contents, with cinnamaldehyde and benzaldehyde being the major constituents [26]. The antioxidant activities of the extracts may be attributable to the capacity of these phenolic compounds to scavenge reactive oxygen species (ROS), thereby neutralizing free radicals as superoxide anions and hydroxyls [48,49].

### 3.2. Essential Oils

In this regard, Tung and colleagues analyzed the anti-inflammatory effect of essential oils from leaves of *C. osmophloeum* on NO production in LPS-activated RAW 264.7 macrophages. NO is endogenously produced from L-Arginine by Nitric Oxide Synthase (NOS) enzyme in various animal tissues. At low concentrations, NO contributes to the regulation of homeostasis; however, excessive NO production is closely associated with inflammatory conditions. The inducible form of NOS (iNOS) can be activated in various cells, such as macrophages, hepatocytes, and Kupffer cells, leading to the production of high concentrations of NO after exposure to LPS from gram-negative bacteria, which triggers tissue damage, cytotoxicity, and inflammation [50]. The authors reported that essential oils from *C. osmophloeum* of cinnamaldehyde or mixed chemotypes strongly inhibited NO production, with the inhibitory effect attributed to *trans*-cinnamaldehyde and τ-cadinol/α-cadinol, respectively [1]. These findings are supported by a previous study by the same research group, which demonstrated that essential oil from twigs of *C. osmophloeum* and its major constituents, such as *trans*-cinnamaldehyde, caryophyllene oxide, L-borneol, L-bornyl acetate, eugenol, β-caryophyllene, *E*-nerolidol, and cinnamyl acetate inhibited NO and prostaglandin E_2_ (PGE_2_) production in LPS-activated RAW 264.7 macrophages [4], highlighting *C. osmophloeum* as a source of compounds for the treatment and prevention of diseases associated with inflammation.

Posteriorly, Hsu and colleagues [2] conducted a phytochemical analysis and evaluated the antioxidant activities of the essential oil from *C. osmophloeum* and its constituents. The authors isolated essential oils of the cinnamaldehyde and camphor chemotypes, where their major compounds *trans*-cinnamaldehyde and D-(+)-camphor, respectively, exhibited in vivo antioxidant activities against juglone-induced oxidative stress in *Caenorhabditis elegans*. This was evidenced by the absence of negative effects on the worms, including survival, growth rate, progeny production, body length, or morphological changes [2]. The researchers attributed the mechanisms underlying the activities of these compounds to their ability to induce the expression of superoxide dismutase-4 (SOD-4) and glutathione S-transferase-4 (GST-4) genes, highlighting their potential as nutraceuticals or antioxidant agents for mitigating injury by free radicals.

In another study, Chao and colleagues [31] identified 21 compounds in the essential oil from *C. osmophloeum*, with the major constituents being monoterpenes and sesquiterpenes, secondary metabolites known for their anti-inflammatory activities [51]. Macrophages play crucial roles in immunomodulation, phagocytosis, and antigen presentation in cell-mediated inflammatory response. After exposure to inflammatory stimuli, these cells secrete a series of pro-inflammatory cytokines, such as IL-1β, IL-6, and TNF-α, which are key agents in the immune response [52,53]. Essential oils from several plant families, such as Geraniaceae, Anacardiaceae, Annonaceae, and Umbelliferae, have been studied for their ability to modulate inflammation via the inhibition of pro-inflammatory cytokines [54]. In this line, the data revealed the inhibitory capacity of the essential oil on pro-IL-1β expression in LPS-stimulated cells. Furthermore, the essential oil from *C. osmophloeum* reduced the production of IL-1β and IL-6, but not TNF-α, suggesting a modulatory effect on the immune response and offering an alternative approach to controlling the inflammatory response in diseases [31].

Essential oil from the linalool chemotype of *C. osmophloeum* demonstrated an anti-inflammatory effect in a murine model of sepsis [30]. Approximately 11 million deaths per year are attributed to sepsis, with septic shock being the most severe form, leading to the death of at least 40% of affected individuals, making it a challenging condition to manage [55,56]. Sepsis induces a rapid immune response due to the action of endotoxins, resulting in liver dysfunction and injury, acute kidney injury, endothelial damage, and altered coagulation [57]. Two signaling pathways have been proposed as potential targets for treatment: toll-like receptor 4 (TLR4) and the Nucleotide-binding and oligomerization domain (NOD)-like receptor family, particularly NLRP3. The TLR4/myeloid differentiation factor 2 (MD2) complex on host cells is the primary receptor activated by endotoxins [58]. After interaction with endotoxins, the complex binds to its adaptor protein, myeloid differentiation primary response gene (88) (MyD88), followed by NF-κB activation, leading to NO production and the secretion of proinflammatory cytokines, such as TNF-α and IL-1β [59]. On the other hand, NLRP3 activation promotes assembling with apoptosis-associated speck-like protein (ASC) and caspase-1, forming the NLRP3 inflammasome, responsible for producing IL-1β and interleukin-18 (IL-18) [60]. Although endotoxins are not direct ligands for NLRP3, there is some evidence suggesting the involvement of this cytosolic pattern recognition receptor in endotoxin-induced inflammation through its association with TLR4 activation by damage-associated molecular pattern molecules (DAMPs). Furthermore, NF-κB can increase NLRP3 expression, thus promoting the activation of the NLRP3 inflammasome by DAMPs [61].

In this context, a research group reported that the essential oil from the *C. osmophloeum* linalool chemotype significantly reduced the peripheral levels of TNF-α, IL-1β, IL-18, IFN-γ, and NO, and inhibited the expression of TLR4, MyD88, MD2, ASC, caspase-1, and NLRP3. Additionally, it inhibited NF-κB activation and caspase-1 activity in the small intestine, providing strong evidence for the protective effect of this essential oil in endotoxin-induced systemic inflammatory response, which is closely associated with the suppression of the TLR4 and NLRP3 signaling pathways [30].

Lee and colleagues [29] demonstrated that the essential oil of *C. osmophloeum* also exhibits antidiabetic activity, improving pancreatic function, oxidative imbalance, and inflammatory markers in rodents with streptozotocin (STZ)-induced diabetes. The authors showed that linalool, the major component of the essential oil, effectively reduced fasting blood glucose and fructosamine, while elevating plasma and pancreatic insulin levels under fasting conditions [29]. These data are supported by previous studies in which phenolic compounds from essential oils exhibited antidiabetic effects by improving serum glucose, triglycerides, total cholesterol, and insulin activity [62,63,64].

**Figure 5:**
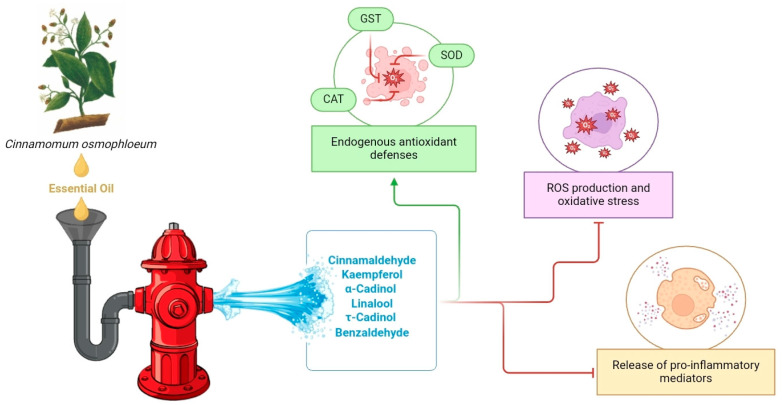
Antioxidant and anti-inflammatory effects of *C. osmophloeum* essential oils and their constituents. Compounds extracted from the leaves and bark of *C. osmophloeum* improve endogenous antioxidant defenses (green line), reduce ROS production through their antioxidant capacity, and suppress the release of pro-inflammatory mediators (red line). Abbreviations: CAT, catalase; GST, glutathione S-transferase; ROS, reactive oxygen species; SOD, superoxide dismutase.

Oxidative stress plays a critical role in impaired β-cell function [65], leading to cellular damage due to an imbalance between antioxidant agents and lipid peroxidation, which is largely caused by an excess of ROS and reactive nitrogen species (RNS), both of which are elevated in diabetes [62]. In this regard, Lee and colleagues showed that the essential oil from *C. osmophloeum* improved pancreatic levels of thiobarbituric acid-reactive substances (TBARS) and the activities of superoxide dismutase (SOD) and glutathione reductase (GR). Moreover, NO, a key mediator in inflammation whose synthesis is markedly increased through elevated iNOS expression induced by pro-inflammatory cytokines and ROS, has been implicated in β-cell damage in diabetes [66]. The authors also showed that treatment with the essential oil ameliorated pancreatic levels of NO, IL-1β, and TNF-α in rats with STZ-induced diabetes. These findings suggest that the essential oil of *C. osmophloeum* may have a protective effect on pancreatic β-cells, improving antioxidant capacity, modulating the inflammatory response, and reducing oxidative stress in diabetic rats [29]. Figure 5 illustrates the potential beneficial effects of *C. osmophloeum* essential oils and their constituents.

### 3.3. Chemical Constituents

In addition to the effects observed with the use of essential oils and extracts from *C. osmophloeum*, some of its constituents also stand out for their broad spectrum of biological activities. Liu and colleagues [67] evaluated the anti-inflammatory effect of the cinnamaldehyde from *C. osmophloeum* in a DSS-induced colitis model in mice. The results showed that oral administration of this compound attenuated the expression of key inflammatory markers, such as TNF-α, monocyte chemoattractant protein-1 (MCP-1), myeloperoxidase (MPO), and cyclooxygenase-2 (COX-2), thereby reducing dextran sulfate sodium (DSS)-induced colonic injury. A detailed analysis revealed that cinnamaldehyde reduced IL-1β levels in the colon of the animals, associated with a decrease in the colonic expression of NLRP3 and ASC [67]. Upon activating, the NLRP3 inflammasome, which composes a sensor (NLRP3), an adaptor molecule (ASC), and an effector protein (pro-caspase-1), triggers the cleavage of pro-caspase-1 into caspase-1, resulting in the release of biologically active IL-1β and IL-18 [68]. Thus, it is reasonable to conclude that cinnamaldehyde exerts potent anti-inflammatory action in the colonic mucosa by inhibiting the activation of the NLRP3 inflammasome.

To further investigate the role of the NLRP3 inflammasome in the anti-inflammatory effects of cinnamaldehyde, the authors used adenosine triphosphate (ATP)-stimulated J774A.1 macrophage in vitro. Consistent with previous findings, they observed that cinnamaldehyde effectively suppressed IL-1β levels and downregulated caspase-1 expression [67]. Similar results were demonstrated by Hua et al. (2024), where cinnamaldehyde reduced IL-1β, IL-18, and caspase-1 levels in a dose-dependent manner in *Shigella sonnei*-stimulated J774A.1 macrophage [69]. Interestingly, under the experimental conditions tested, cinnamaldehyde did not affect the secretion of TNF-α and IL-6, which are NLRP3 inflammasome-independent cytokines, strongly suggesting that the anti-inflammatory effect of cinnamaldehyde involves NLRP3 inhibition. It was also investigated whether the effect of cinnamaldehyde required the involvement of another multiprotein complex, the NLRC4 inflammasome. However, this hypothesis was discarded by the researchers, since cinnamaldehyde reduced IL-1β levels even in NLRC4-knockout THP-1 macrophages. This suggests that cinnamaldehyde requires the direct involvement of the NLRP3 inflammasome for its biological activity during inflammation.

It is known that endotoxins, including LPS, bind to membrane receptors, primarily TLRs, to activate the NLRP3 inflammasome [70]. Thus, TLRs stimulate priming signals, including mitogen-activated protein kinases (MAPKs) and NF-κB, which lead to the transcriptional expression of NLRP3 and proIL-1β, the precursor of IL-1β [71]. In this context, Hua et al. (2024) investigated whether pre-incubation with cinnamaldehyde affects NLRP3 inflammasome priming signals using LPS-stimulated J774A.1 macrophages [69]. The authors found that cinnamaldehyde did not interfere with the phosphorylation of MAPKs (Extracellular signal-regulated kinase 1/2 (ERK1/2), C-Jun N-terminal kinase 1/2 (JNK1/2), or p38), nor did it alter the phosphorylation of NF-κB p65, inhibitor of nuclear factor kappa B-α (IκB-α), and inhibitory-κB kinase-α/β (IKK-α/β). These results suggest that cinnamaldehyde affects NLRP3 activation independently of TLR4 and its priming signals. These data agree with the findings of Liu and colleagues (2024), who demonstrated that cinnamaldehyde does not affect NLRP3 and ASC expression in LPS-activated macrophages [67].

Another potential hypothesis to explain cinnamaldehyde-mediated NLRP3 inhibition involves the induction of autophagy. The autophagy process can eliminate inflammasome components or their endogenous activators, such as ROS-producing damaged mitochondria, thereby resulting in NLRP3 suppression [72]. This hypothesis is supported by Hua et al. (2024), who demonstrated that cinnamaldehyde increased the levels of microtubule-associated protein 1A/1B-light chain 3-II (LC3-II), autophagy-related protein 5 (ATG5), and p62 in J774A.1 macrophages, which are markers of autophagy [69]. To test whether cinnamaldehyde-induced autophagy is related to NLRP3 inflammasome suppression, the authors used 3-MA, an autophagy inhibitor, and LC3-knockout J774A.1 macrophages. The results revealed that IL-1β production was increased in both experimental conditions, even in the presence of cinnamaldehyde, indicating that the disruption of autophagy compromises the inhibitory effect of cinnamaldehyde on the NLRP3 inflammasome. Liu et al. (2024) also obtained similar results, further supporting the hypothesis that cinnamaldehyde inhibits NLRP3 inflammasome activity, at least partially, through autophagy induction [67].

On the other hand, Lee and colleagues [7] investigated the effects of cinnamaldehyde and linalool on TLR4- and NLRP3-mediated signaling pathways in vivo. They demonstrated that both compounds reduced nuclear NF-κB levels and prevented caspase-1 activation in the spleen and mesenteric lymph nodes of endotoxin-injected mice [7], which are downstream molecules of TLR4 and NLRP3 signaling, respectively. The reduction in cytokines related to these two pathways, such as TNF-α and IFN-γ (for TLR4), and IL-1β and IL-18 (for NLRP3), further supports this hypothesis. Additionally, cinnamaldehyde and linalool reduced the endotoxin-induced expression of TLR4, MD2, and MyD88, as well as suppressing NLRP3, ASC, and caspase-1 expression, suggesting that these compounds may regulate both pathways independently to promote their anti-inflammatory effects.

Although cinnamaldehyde does not appear to act through TLRs to suppress NLRP3 activation, it is relevant to study its effects on the TLRs-mediated signaling pathway, as these receptors regulate the production of pro-inflammatory cytokines, contributing significantly to the initiation and progression of inflammatory diseases [73]. In this line, Chao and colleagues [74] investigated the effect of cinnamaldehyde on the J774A.1 macrophage stimulated with ligands of different TLRs receptors. This compound was able to reduce the levels of TNF, IL-6, and IL-1, and the expression of proIL-1 in macrophages activated by LPS, a TLR4 ligand. These results were also observed in two other cell lines: (1) human THP-1 monocytes and (2) human blood monocyte-derived primary macrophages, demonstrating that cinnamaldehyde has an inhibitory effect on cytokine production that is not cell-type-specific. Cinnamaldehyde also reduced TNF-α and IL-1β levels in macrophages stimulated by lipoteichoic acid (LTA), a TLR2 ligand, but had no effect on cytokine production induced by polyinosinic-polycytidylic acid (poly I:C), a TLR3 ligand [74].

Each TLR subtype has a specific recognition pattern. Some TLRs, such as TLR2, TLR4, and TLR5, are located on the cell surface, while other receptors, including TLR3 and TLR7, are expressed in intracellular compartments called endosomes [73]. The regulation of the TLR4/cluster of differentiation 14 (CD14) complex, as well as TLR2, both at the transcriptional and translational levels, appears to be important in modulating the systemic inflammatory response, mainly mediated by macrophages [75]. In this line, Chao and colleagues [74] investigated whether the inhibitory effects of cinnamaldehyde on TLR-mediated cytokine production involve blocking LPS binding to the TLR4/CD14 complex or altering the expression of these proteins on the cell surface of J774A.1 macrophage, thereby decreasing receptor availability. However, the results showed that cinnamaldehyde did not interfere with LPS binding to the TLR4/CD14 complex, nor did it affect the expression of TLR4 or CD14 [74]. Taken together, these results suggest that cinnamaldehyde does not act as an LPS antagonist, but its effect appears to involve the inhibition of the downstream signaling process following LPS binding to TLR4/CD14.

The MAPK pathway is a crucial downstream signaling cascade of TLR4 that contributes to the regulation of LPS-stimulated cytokine production [76]. In this line, Chao et al. (2008) demonstrated that cinnamaldehyde effectively reduced phosphorylation of ERK1/2 and JNK1/2 in LPS-stimulated macrophages, suggesting that MAPK inhibition could be one of the mechanisms by which cinnamaldehyde exerts its anti-inflammatory effects [73]. Furthermore, cinnamaldehyde suppressed ROS production, a key mediator of the inflammatory response, primarily through TLR4 activation. The inhibition of ROS production can reduce TLR4-mediated pro-inflammatory cytokine expression, thereby suppressing local and systemic inflammation [77]. ROS plays a crucial role in activating MAPKs through different mechanisms, such as direct signaling, activation of growth factor receptors, or inactivation of proteins that dephosphorylate MAPKs [78], which may help explain the inhibitory effects of cinnamaldehyde on MAPKs.

Other constituents of *C. osmophloeum* leaves have demonstrated significant effects on LPS-induced inflammation. In a study by Fang and colleagues [33], the biological activity of four flavonol glycosides (**1**, **2**, **3** and **4**) was evaluated. Using murine peritoneal macrophages stimulated with LPS plus IFN-γ, the authors demonstrated that these compounds reduced nitrite concentrations, a marker for NO production, in a dose-dependent manner, and suppressed the release of TNF-α and IL-12 [33]. These results are aligned with the literature, as flavonol glycosides from other plant species have also demonstrated anti-inflammatory, antinociceptive [79], antimicrobial, and antioxidant activities [80], thus presenting a broad spectrum of biological activity. Although all the flavonol glycosides had significant effects, compound **3** exhibited the most effective results. In another experimental protocol, the authors exposed the cells to LPS for 24 h to induce iNOS expression before adding the flavonol glycosides. The results indicated that compounds **3** and **4** reduced nitrite concentrations, suggesting that the anti-inflammatory effects of these compounds can control the enzymatic synthesis of NO [33]. Regulation of NO production via the inhibition of iNOS enzymatic activity is a crucial strategy for treating inflammatory diseases [81], highlighting the potential anti-inflammatory effect of constituents of *C. osmophloeum*.

NO is a molecule with dual effects, exerting multiple physiological effects, such as fluid and electrolyte transport, vascular tone, blood pressure, and platelet aggregation, while also serving as a well-known mediator of inflammation and being implicated in several inflammatory diseases [82]. Endogenous NO is produced by three NOS isoforms: neuronal NOS (nNOS) and endothelial NOS (eNOS), which produce NO constitutively for signaling purposes, and iNOS, which is expressed following induction by inflammatory mediators [83]. These enzymes are highly expressed in renal tissue, where a proper balance of NO production is essential for normal kidney function. The dysregulation of NO pathways can contribute to various renal diseases, such as diabetic nephropathy and acute kidney injury [84,85].

This gasotransmitter binds to the heme group of soluble guanylate cyclase (sGC), activates the enzyme, and triggers the conversion of guanosine triphosphate (GTP) into cyclic guanosine monophosphate (cGMP), culminating in the activation of cGMP-dependent protein kinase (PKG), which plays a role in the regulation of homeostasis across various biological systems [85]. In this context, Huang and colleagues [86] evaluated the effect of cinnamaldehyde on the NO/cGMP/PKG pathway using human renal proximal tubular cells (HK-2) treated with advanced glycation end products (AGEs). AGEs are compounds formed through the non-enzymatic condensation of carbonyl groups from reducing sugars with free amine groups of nucleic acids, proteins, or lipids, and are associated with the development of diabetic nephropathy [87]. The study observed that cinnamaldehyde reversed the inhibition of AGE-induced NO production in a dose-dependent manner, while also restoring cGMP synthesis and PKG activation [86]. These results suggest that the NO/cGMP/PKG pathway plays a crucial role in the cinnamaldehyde-mediated protection of renal cells. Additionally, Western blot analysis revealed that cinnamaldehyde reversed the inhibitory effect of AGEs on the protein levels of iNOS and eNOS, strongly suggesting that the action of cinnamaldehyde on AGE-induced effects may involve NOS-dependent mechanisms, thereby contributing to the regulation of sustained generation of NO [87].

In another experimental design, Huang and colleagues [86] also examined the effects of cinnamaldehyde on the activation of the JAK/STATs pathway in AGE-activated HK-2 cells. This signaling pathway plays a crucial role in diabetic nephropathy, where it can be activated by high glucose levels or derived compounds, including AGEs [88]. In this line, cinnamaldehyde was effective in reducing the phosphorylation of Janus kinase 2 (JAK2), signal transducer and activator of transcription 1 (STAT1), and signal transducer and activator of transcription 3 (STAT3), which was increased by AGEs, showing results like those obtained with AG490, a JAK2 inhibitor, but without affecting the expression levels of these proteins [87]. Further, analysis also revealed that cinnamaldehyde significantly reversed the AGE-induced inhibition of suppressor of cytokine signaling-3 (SOCS-3) expression, a protein that inhibits JAK2 catalytic activity and leads to the inactivation of the JAK2/STATs pathway. Taken together, these results suggest that the JAK2-STAT1/STAT3 pathway may play a crucial role in mediating the effects of AGEs on renal cells, with cinnamaldehyde emerging as an effective strategy to attenuate the activation of this signaling cascade.

Although cinnamaldehyde stands out as one of the major constituents of *C. osmophloeum*, other compounds may have potential protective effects, especially through anti-inflammatory mechanisms. In this regard, Tung and colleagues [89] investigated the hepatoprotective effect of aromadendrene, τ-cadinol, α-cadinol, and *trans*-cinnamaldehyde in a model of LPS/D-galactosamine (D-GalN)-induced acute hepatitis in mice. Before conducting in vivo tests, the authors evaluated whether these four compounds affected NO production in LPS-activated RAW 264.7 macrophages. This analysis is important due to the central role of macrophages in developing hepatic inflammation, as they secrete inflammatory mediators in response to injury or infection [90]. The results showed that all compounds inhibited NO production dose-dependent, with α-cadinol being the most effective [89]. Further analysis revealed that α-cadinol downregulated iNOS expression in these cells. Additionally, the authors showed that α-cadinol suppresses the degradation of the NF-κB inhibitor IκB-α by inhibiting the activation of IKK-α/β, which functions to degrade IκB-α and release NF-κB. This prevents the release and translocation of NF-κB to the nucleus, blocking its role in the transcription of pro-inflammatory genes, thereby contributing to the reduction in iNOS expression.

In the acute hepatitis model, Tung and colleagues showed that the four compounds tested were able to reduce serum levels of alanine transaminase (ALT) and aspartate transferase (AST), key markers of liver injury, in addition to attenuating serum levels of TNF-α and IL-6 in mice injected with LPS/D-GalN [89]. As observed in vitro, α-cadinol was the most effective in reducing the levels of these pro-inflammatory cytokines. The histopathological analysis also revealed that the four compounds attenuated the infiltration of inflammatory cells and liver tissue destruction induced by LPS/D-GalN administration. Notably, Western blot results revealed that α-cadinol and its isomer, τ-cadinol, decreased hepatic expression of cleaved caspase-3 and poly-ADP ribose polymerase (PARP), an effector of the apoptotic pathway and its substrate, respectively, suggesting that the inhibition of apoptosis may also be an important mechanism associated with the protective effects of constituents from *C. osmophloeum*.

The main articles showing the biological activities of the crude extracts, essential oils, and chemical constituents of *C. osmophloeum* are summarized in Table 3.

**Figure 6:**
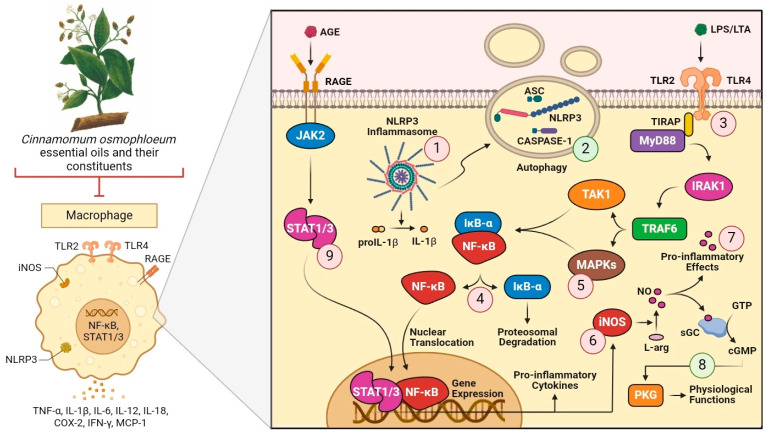
Cellular mechanisms involved in the beneficial effects of *C. osmophloeum* essential oils and their constituents. Abbreviations: AGE, advanced glycation end products; ASC, apoptosis-associated speck-like protein containing a caspase recruitment domain; cGMP, cyclic guanosine monophosphate; COX-2, cyclooxygenase-2; GTP, guanosine triphosphate; IκB-α, NF-κB inhibitor alpha; IFN-γ, interferon gamma; IL, interleukin; iNOS, inducible nitric oxide synthase; JAK2, Janus kinase 2; L-arg, L-arginine; LPS, lipopolysaccharide; LTA, lipoteichoic acid; MAPKs, mitogen-activated protein kinases; MCP-1, monocyte chemoattractant protein-1; MyD88, myeloid differentiation primary response 88; NF-κB, nuclear factor kappa B; NLRP3, NOD-like receptor protein 3; NO, nitric oxide; PKG, protein kinase G; RAGE, receptor for advanced glycation end products; sGC, soluble guanylate cyclase; STAT1/3, signal transducer and activator of transcription 1/3; TLR2, toll-like receptor type 2; TLR4, toll-like receptor type 4; TNF-α, tumor necrosis factor alpha.

Figure 6 summarizes the main cellular mechanisms involved in the biological activities of *C. osmophloeum* and its constituents. The compounds can reduce the production of inflammatory cytokines, such as IL-1β, by inhibiting the activation of the NLRP3 inflammasome (**1**). One of the possible mechanisms of NLRP3 inhibition is the induction of autophagy, which would contribute to the exocytosis of inflammasome components (**2**). In addition, they can also suppress the activation of TLR2 and TLR4 (**3**), culminating in the blockade of the dissociation of IκB-α and NF-κB and their translocation to the nucleus (**4**). The involvement of MAPKs (**5**) and iNOS (**6**) inhibition has also been demonstrated as important anti-inflammatory mechanisms. However, although the benefits of NO inhibition have been shown to prevent its pro-inflammatory effects (**7**), the NO/cGMP/PKG pathway can be stimulated to promote physiological functions and protection (**8**). Finally, the compounds also inhibit the activation of the JAK2/STAT1/3 pathway (**9**), which contributes to the reduction in the production of pro-inflammatory mediators.

## 4. Conclusions and Future Perspectives

This review presents the most recent research on the inflammatory response and oxidative imbalance of essential oils, extracts, and chemical constituents of *C. osmophloeum*. The major secondary metabolites identified in *C. osmophloeum* were essential oils and flavonol glycosides. Regarding essential oils, the cinnamaldehyde chemotype was the main detected in *C. osmophloeum* leaves. Indigenous cinnamon contains several biologically active compounds, and given its popular use, major compounds with a well-known structure emerge as promising candidates for drugs or supplements, particularly as they facilitate the isolation and large-scale production of these compounds. Despite the extensive in vitro and in vivo data supporting the therapeutical potential of essential oils from *C. osmophloeum* and their constituents, further human studies are required to confirm its clinical applicability. Furthermore, there is a need for investigation into the therapeutic safety of these molecules to rule out possible toxic effects and determine appropriate therapeutic doses. Overall, *C. osmophloeum* appears to be a promising source of compounds capable of improving the health and well-being of patients with inflammatory diseases and imbalance oxidative.

## Figures and Tables

**Table 1:** Chemical composition of the essential oils from *C. osmophloeum* and their relative contents * (%) (in some studies, different *C. osmophloeum* specimens were evaluated).

Plant Material and Oil Yield (% or mL/kg)	Chemical Composition and Their Relative Contents (%)	Reference
Cinnamaldehyde type		
Leaves (7.93 mL/kg)	Cinnamon B—cinnamaldehyde (76.0%), geranyl acetate (3.88%), benzaldehyde (3.20%), geraniol (2.96%), eugenol (1.05%), cinnamyl acetate (0.51%), coumarin (0.26%), and linalool (0.24%)	[12]
Leaves (7.93 mL/kg)	Cinnamon B—cinnamaldehyde (76.0%), geranyl acetate (3.88%), benzaldehyde (3.20%), geraniol (2.96%), geraniol (1.05%), cinnamyl acetate (0.51%), coumarin (0.26%), and linalool (0.40%)	[13]
Leaves (1.02%)	Cinnamon A—*trans*-cinnamaldehyde (60.22%), cinnamyl acetate (7.54%), β-caryophyllene (4.71%), bornyl acetate (4.69%), benzenepropanal (4.50%), caryophyllene oxide (3.59%), eugenol (2.40%), anethole (1.78%), rimuen (1.75%), benzaldehyde (1.74%), verticiol (1.11%), *cis*-cinnamaldehyde (1.10%), τ-cadinol (0.89%), α-caryophyllene (0.78%), guaiol (0.72%), γ-elemene (0.68%), β-cadinene (0.62%), α-cubebene (0.57%), and isoledene (0.54%)	[15]
Leaves (1.11%)	Cinnamon G—*trans*-cinnamaldehyde (85.32%), benzenepropanal (3.39%), benzaldehyde (1.75%), anethole (1.45%), *cis*-cinnamaldehyde (1.42%), caryophyllene oxide (0.68%), β-caryophyllene (0.66%), eugenol (0.63%), β-cadinene (0.61%), terpinene-4-ol (0.44%), cinnamyl acetate (0.43%), α-cubenene (0.41%), copaene (0.34%), γ-elemene (0.32%), 4-allyphenol (0.31%), *p*-cymene (0.26%), camphor (0.26%), isoledene (0.24%), γ-murrolene (0.20%), and bornyl acetate (0.17%)	[15]
Leaves (0.89%)	Cinnamon H—*trans*-cinnamaldehyde (74.57%), benzenepropanal (8.41%), benzaldehyde (5.92%), eugenol (3.02%), anethole (2.75%), cinnamyl acetate (1.89%), *cis*-cinnamaldehyde (1.32%), β-caryophyllene (0.80%), caryophyllene oxide (0.79%), and γ-elemene (0.54%)	[15]
Leaves (1.1%)	Cinnamon 1—*cis*-cinnamaldehyde (85.3%), benzenepropanal (3.4%), anethole (1.5%), *trans*-cinnamaldehyde (1.4%), β-caryophyllene (0.7%), caryophyllene oxide (0.7%), β-cadinene (0.6%), eugenol (0.6%), α-cubebene (0.4%), terpinene-4-ol (0.4%), cinnamyl acetate (0.4%), camphor (0.3%), *p*-cymene (0.3%), T-muurolene (0.2%), isoledene (0.2%), and bornyl acetate (0.2%)	[11]
Leaves (6.92 mL/kg)	Cinnamon B—cinnamaldehyde (81.08%), geranyl acetate (3.01%), benzaldehyde (2.88%), geraniol (2.58%), cinnamyl acetate (1.11%), eugenol (0.95%), coumarin (0.50%), and linalool (0.12%)	[20]
Leaves (7.93 mL/kg)	Cinnamon C—cinnamaldehyde (76.0%), geranyl acetate (3.88%), benzaldehyde (3.20%), geraniol (2.96%), eugenol (1.05%), τ-cadinol (0.9%), guaiol (0.7%), cinnamyl acetate (0.51%), coumarin (0.26%), and linalool (0.24%)	[20]
Leaves (0.9%)	Cinnamon CO1—*trans*-cinnamaldehyde (60.3%), cinnamyl acetate (7.4%), bornyl acetate (4.7%), β-caryophyllene (4.7%), benzenepropanal (4.5%), caryophyllene oxide (3.6%), eugenol (2.4%), anethole (1.8%), benzaldehyde (1.7%), rimuen (1.4%), *cis*-cinnamaldehyde (1.1%), α-caryophyllene (0.8%), γ-elemene (0.7%), α-cubebene (0.6%), β-cadinene (0.6%), and isoledene (0.5%)	[16]
Leaves (1.1%)	Cinnamon CO7—*trans*-cinnamaldehyde (81.0%), benzenepropanal (6.3%), benzaldehyde (3.8%), eugenol (2.8%), anethole (1.6%), *cis*-cinnamaldehyde (1.0%), cinnamyl acetate (0.9%), β-caryophyllene (0.8%), bornyl acetate (0.7%), caryophyllene oxide (0.4%), 4-allyphenol (0.4%), and δ-cadinene (0.3%)	[16]
Leaves (1.7%)	Cinnamon CO8—*trans*-cinnamaldehyde (76.7%), benzenepropanal (5.4%), cinnamyl acetate (5.4%), benzaldehyde (3.4%), eugenol (2.5%), anethole (1.6%), β-caryophyllene (1.3%), *cis*-cinnamaldehyde (1.0%), bornyl acetate (1.0%), caryophyllene oxide (0.8%), 4-allyphenol (0.6%), and δ-cadinene (0.4%)	[16]
Leaves (no information)	Cinnamon A—*trans*-cinnamaldehyde (91.15%), terpin-4-ol (2.94%), benzenepropanal (1.52%), bornyl acetate (1.51%), eugenol (1.47%), methyl chavicol (1.45%), caryophyllene (0.71%), *cis*-cinnamaldehyde (0.66%), α-thujene (0.42%), α-pinene (0.35%), benzaldehyde (0.61%), 1,8-cineole (0.60%), cinnamyl acetate (0.57%), caryophyllene oxide (0.40%), D-limonene (0.34%), (+)-4-carene (0.26%), copaene (0.26%), α-phellandrene (0.21%), linalool (0.17%), camphene (0.16%), *p*-cymene (0.11%), and β-humulene (0.11%)	[22]
Leaves (1.11%)	Cinnamon A—trans-cinnamaldehyde (91.32%), benzenepropanal (3.18%), 4-allylanisole (1.42%), L-bornyl acetate (0.59%), and cinnamyl acetate (0.44%)	[14]
Leaves (1.02%)	*trans*-cinnamaldehyde (79.85%), benzenepropanal (7.0%), benzaldehyde (5.35%), *cis*-cinnamaldehyde (1.91%), *p*-allylanisole (1.61%), eugenol (1.13%), L-bornyl acetate (1.11%), cinnamyl acetate (0.62%), 4-allylphenol (0.22%), β-caryophyllene (0.19%), and α-cubebene (0.17%)	[17]
Leaves (3.3%)	*trans*-cinnamaldehyde (74.16%), cinnamyl acetate (20.61%), 3-pheayl pionaldehyde (1.29%), benzaldehyde (1.24%), *cis*-cinnamaldehyde (0.89%), eugenol (0.81%), isobornyl acetate (0.52%), α-pinene (0.23%), camphene (0.15%), β-pinene (0.10%)	[21]
Leaves (1.1%)	Cinnamon COB—*trans*-cinnamaldehyde (91.09%), benzenepropanal (3.30%), 4-allylanisole (1.43%), benzaldehyde (1.19%), *cis*-cinnamaldehyde (0.88%), L-bornyl acetate (0.56%), caryophyllene oxide (0.48%), terpinen-4-ol (0.46%), and eugenol (0.28%)	[18]
Leaves (no information)	Cinnamon COA—*trans*-cinnamaldehyde (72.93%), cinnamyl acetate (8.82%), 3-phenyl propionaldehyde (5.35%), L-bornyl acetate (3.84%), β-caryophyllene (2.74%), eugenol (1.2%), caryophyllene oxide (1.06%), 4-allylanisole (0.8%), α-humulene (0.42%), benzaldehyde (0.38%), *cis*-cinnamaldehyde (0.37%), α-copaene (0.25%), δ-cadinene (0.23%), linalool (0.22%), α-terpineol (0.18%), chavicol (0.13%), and L-borneol (0.10%)	[1]
Leaves (no information)	Cinnamon COB—*trans*-cinnamaldehyde (62.64%), cinnamyl acetate (9.54%), 3-phenyl propionaldehyde (6.72%), L-bornyl acetate (4.83%), benzaldehyde (3.41%), β-caryophyllene (4.1%), eugenol (1.57%), caryophyllene oxide (1.25%), 4-allylanisole (1.13%), α-pinene (0.5%), α-copaene (0.44%), α-humulene (0.4%), camphene (0.4%), *cis*-cinnamaldehyde (0.35%), β-pinene (0.33%), δ-cadinene (0.27%), limonene (0.25%), α-terpineol (0.22%), chavicol (0.16%), mesitylene (0.14%), and L-borneol (0.11%)	[1]
Leaves (no information)	Cinnamon COC—*trans*-cinnamaldehyde (77.21%), cinnamyl acetate (11.57%), 3-phenyl propionaldehyde (3.35%), benzaldehyde (2.77%), L-bornyl acetate (1.11%), 4-allylanisole (0.73%), *cis*-cinnamaldehyde (0.53%), β-caryophyllene (0.41%), eugenol (0.29%), δ-cadinene (0.23%), caryophyllene oxide (0.18%), α-pinene (0.17%), camphene (0.13%), α-copaene (0.13%), and β-pinene (0.12%)	[1]
Leaves (1.1%)	*trans*-cinnamaldehyde (85.55%), benzenepropanal (5.92%), benzaldehyde (4.39%), 4-allylanisole (1.39%), eugenol (1.0%), L-bornyl acetate (0.86%), *trans*-cinnamaldehyde (0.62%), and geranyl acetate (0.27%)	[19]
Leaves (1.28%)	Cinnamon Y3—*trans*-cinnamaldehyde (73.99%), *trans*-cinnamyl acetate (12.61%), 3-phenyl propionaldehyde (3.69%), L-bornyl acetate (1.19%), 4-allylanisole (0.81%), *trans*-β-caryophyllene (0.49%)	[2]
Leaves (no information)	Cinnamon A—*trans*-cinnamaldehyde (88.74%), benzenepropanal (4.47%), benzaldehyde (3.47%), 4-allylanisole (0.94%), *cis*-cinnamaldehyde (0.64%), eugenol (0.43%), L-bornyl acetate (0.27%), *trans*-cinnamyl acetate (0.21%), and *trans*-β-caryophyllene (0.13%)	[23]
Leaves (no information)	Cinnamon B—*trans*-cinnamaldehyde (95.25%), benzaldehyde (1.56%), benzenepropanal (0.96%), *cis*-cinnamaldehyde (0.72%), *p*-cymene (0.63%), L-bornyl acetate (0.44%), 4-allylanisole (0.26%), *trans*-β-caryophyllene (0.17%), caryophyllene oxide (0.17%), and α-pinene (0.10%)	[23]
Leaves (no information)	Cinnamon C—*trans*-cinnamaldehyde (87.25%), benzaldehyde (3.63%), benzenepropanal (2.97%), L-bornyl acetate (0.95%), *trans*-cinnamyl acetate (0.74%), eugenol (0.65%), *cis*-cinnamaldehyde (0.61%), α-pinene (0.57%), *trans*-β-caryophyllene (0.55%), 4-allylanisole (0.35%), camphene (0.32%), δ-cadinene (0.29%), caryophyllene oxide (0.27%), limonene (0.24%), α-copaene (0.23%), β-pinene (0.22%), and α-terpineol (0.10%)	[23]
Leaves (no information)	Cinnamon D—*trans*-cinnamaldehyde (81.43%), benzaldehyde (5.91%), benzenepropanal (3.88%), *trans*-cinnamyl acetate (3.31%), L-bornyl acetate (1.27%), 4-allylanisole (0.94%), *trans*-β-caryophyllene (0.69%), α-pinene (0.38%), *cis*-cinnamaldehyde (0.34%), spathulenol (0.33%), camphene (0.33%), 1,8-cineole (0.33%), eugenol (0.28%),α-copaene (0.21%), caryophyllene oxide (0.18%), and limonene (0.14%)	[23]
Leaves (no information)	Cinnamon E—*trans*-cinnamaldehyde (68.44%), linalool (14.38%), *trans*-cinnamyl acetate (10.88%), eugenol (1.26%), *cis*-cinnamaldehyde (0.81%), benzaldehyde (0.76%), *trans*-anethole (0.75%), benzenepropanal (0.71%), *trans*-β-caryophyllene (0.58%), δ-cadinene (0.32%), α-cadinol (0.29%), 4-allylanisole (0.25%), δ-cadinol (0.25%), α-terpineol (0.17%), and caryophyllene oxide (0.11%)	[23]
Leaves (2.82%)	*trans*-cinnamaldehyde (70.20%), *trans*-cinnamyl acetate (27.05%), benzenepropanal (0.82%), L-bornyl acetate (0.48%), benzaldehyde (0.45%), *cis*-cinnamaldehyde (0.33%), *trans*-β-caryophyllene (0.18%), 4-allylanisole (0.17%), 2-methyl benzofuran (0.14%), and eugenol (0.1%)	[24]
Leaves (no information)	*trans*-cinnamaldehyde (84.13%), benzaldehyde (7.16%), 3-phenylpropionaldehyde (3.62%), α-pinene (1.09%), L-bornyl acetate (0.92%), camphene (0.79%), methyl chavicol (0.55%), *cis*-cinnamaldehyde (0.50%), β-pinene (0.36%), α-copaene (0.34%), β-caryophyllene (0.29%), and limonene (0.21%)	[25]
Leaves (0.82%)	*trans*-cinnamaldehyde (87.7%), benzaldehyde (7.0%), and cinnamyl acetate (5.3%)	[26]
Cinnamaldehyde/cinnamyl acetate type		
Leaves (1.02%)	Cinnamon B—*trans*-cinnamaldehyde (50.93%), cinnamyl acetate (28.48%), benzenepropanal (3.69%), benzaldehyde (2.07%), rimuen (1.75%), eugenol (1.63%), bornyl acetate (1.61%), verticiol (1.57%), β- caryophyllene (1.09%), caryophyllene oxide (0.91%), τ-cadinol (0.74%), β-cadinene (0.61%), α-cadinol (0.61%), γ-elemene (0.50%), benzyl alcohol (0.44%), anethole (1.25%), *cis*-cinnamaldehyde (1.0%), benzyl alcohol (0.44%), kaur-16-ene (0.39%), labda-8(20),12,14-triene (0.38%), and copaene (0.35%)	[15]
Leaves (2.19%)	Cinnamon C—cinnamyl acetate (44.94%) (49.94%), *trans*-cinnamaldehyde (33.93%), camphene (9.01%), bornyl acetate (1.61%), eugenol (4.59%), benzenepropanal (1.22%), β-caryophyllene (1.07%), benzaldehyde (0.87%), camphor (0.85%), β-cadinene (0.65%), *cis*-cinnamaldehyde (0.62%), α-cadinol (0.51%), τ-cadinol (0.43%), bornyl acetate (0.48%), anethole (0.38%), 4-allyphenol (0.32%), caryophyllene oxide (0.26%), γ-elemene (0.23%), cinnamyl alcohol (0.23%), and α-terpineol (0.21%)	[15]
Leaves (1.0%)	Cinnamon 3—*cis*-cinnamaldehyde (50.9%), cinnamyl acetate (28.5%), benzenepropanal (3.7%), benzaldehyde (2.1%), rimuen (1.8%), verticiol (1.6%), bornyl acetate (1.6%), eugenol (1.6%), anethole (1.3%), β-caryophyllene (1.1%), *trans*-cinnamaldehyde (1.0%), caryophyllene oxide (0.9%), T-cadinol (0.7%), α-cadinol (0.6%), and β-cadinene (0.6%)	[11]
Leaves (1.2%)	Cinnamon CO2 -*trans*-cinnamaldehyde (50.7%), cinnamyl acetate (28.7%), benzenepropanal (3.6%), benzaldehyde (2.2%), rimuen (1.8%), bornyl acetate (1.6%), eugenol (1.6%), verticiol (1.6%), β-caryophyllene (1.1%), caryophyllene oxide (0.9%), τ-cadinol (0.7%), α-cadinol (0.6%), β-cadinene (0.6%), γ-elemene (0.5%), benzyl alcohol (0.4%), labda-8(20),12,14,-triene (0.4%), copaene (0.4%), and kaur-16-ene (0.4%)	[16]
Leaves (2.4%)	Cinnamon CO3—cinnamyl acetate (44.5%), trans-cinnamaldehyde (34.1%), camphene (9.0%), eugenol (4.5%), anethole (1.1%), *cis*-cinnamaldehyde (1.0%), benzenepropanal (1.0%), β-caryophyllene (1.0%), benzaldehyde (0.8%), camphor (0.8%), β-cadinene (0.7%), α-cadinol (0.5%), τ-cadinol (0.4%), bornyl acetate (0.4%), cinnamyl alcohol (0.2%), γ-elemene (0.2%), caryophyllene oxide (0.2%), and α-terpineol (0.2%)	[16]
Leaves (no information)	Cinnamon B—cinnamyl acetate (46.39%), *trans*-cinnamaldehyde (45.59%), *p*-cymene (2.25%), methyl chavicol (2.11%), eugenol (2.03%), terpin-4-ol (1.98%), benzenepropanal (1.39%), bornyl acetate (1.28%), α-terpineol (1.04%), (+)-4-carene (1.01%), neral (0.98%), benzaldehyde (0.74%), α-thujene (0.54%), D-limonene (0.52%), 1,8-cineole (0.42%), caryophyllene (0.42%), *cis*-cinnamaldehyde (0.39%), α-pinene (0.37%), copaene (0.31%), β-humulene (0.23%), linalool (0.23%), β-pinene (0.22%), camphene (0.13%), sabinene (0.12%), and α-phellandrene (0.11%)	[22]
Leaves (1.64%)	Cinnamon B—cinnamyl acetate (56.44%), *trans*-cinnamaldehyde (35.09%), benzenepropanal (1.50%), L-bornyl acetate (0.69%), 4-allylanisole (0.67%), anethole (0.6%), *cis*-cinnamaldehyde (0.5%), and 4-allyphenol (0.3%)	[14]
Leaves (1.64%)	Cinnamon COD—cinnamyl acetate (54.31%), *trans*-cinnamaldehyde (36.50%), benzenepropanal (1.61%), benzaldehyde (1.55%), cinnamyl alcohol (0.97%), 4-allylanisole (0.72%), l-bornyl acetate (0.70%), camphor (0.65%), eugenol (0.57%), *cis*-cinnamaldehyde (0.55%), caryophyllene oxide (0.50%), and δ-cadinene (0.40%)	[18]
Cinnamyl acetate type		
Leaves (1.6%)	Cinnamon 2—cinnamyl acetate (54.4%), 2-methylbenzofuran (14.4%), geranyl acetate (4.4%), caryophyllene (3.7%), τ-cadinol (3.4%), β-cadinene (3.0%), *cis*-cinnamaldehyde (2.9%), δ-cadinene (2.1%), isoledene (1.2%), benzenepropanal (1.2%), bornyl acetate (1.0%), *p*-allylanisole (0.8%), γ-elemene (0.5%), α-muurolene (0.4%), salicylaldehyde (0.4%)	[11]
Leaves (1.6%)	Cinnamon CO9—cinnamyl acetate (54.4%), 2-methylbenzofuran (14.4%), geranyl acetate (4.4%), caryophyllene oxide (3.7%), τ-cadinol (3.4%), β-cadinene (3.0%), *trans*-cinnamaldehyde (2.9%), δ-cadinene (2.1%), isoledene (1.2%), bornyl acetate (1.0%), *p*-allylanisole (0.8%), γ-elemene (0.5%), α-muurolene (0.4%), and salicylaldehyde (0.4%)	[16]
Leaves (1.59%)	Cinnamon E—cinnamyl acetate (60.77%), 2-methylbenzofuran (8.27%), geranyl acetate (4.01%), *trans*-cinnamaldehyde (3.15%), spathulenol (0.99%), L-bornyl acetate (0.94%), and 4-allylanisole (0.92%)	[14]
Leaves (1.59%)	Cinnamon COC—cinnamyl acetate (59.67%), 2-methylbenzofuran (9.32%), geranyl acetate, (4.71%), geranial (3.19%), caryophyllene oxide (3.0%), α-cadinol (2.95%), coumarin (2.67%), τ-cadinol (2.34%), cinnamyl alcohol (2.27%), δ-cadinene (1.43%), cinnamyl formate (1.40%), L-bornyl acetate (1.04%), spathulenol (0.98%), 4-allylanisole (0.76%), salicylaldehyde (0.60%), benzaldehyde (0.49%), and camphor (0.40%)	[18]
Leaves (no information)	Cinnamon COE—cinnamyl acetate e (40.78%), *trans*-cinnamaldehyde (9.09%), caryophyllene oxide (6.2%), τ-cadinol (5.49%), α-cadinol (5.39%), L-bornyl acetate (2.88%), α-copaene (2.3%), δ-cadinene (2.3%), 4-allylanisole (1.69%), coumarin (1.36%), β-caryophyllene (0.96%), benzaldehyde (0.86%), aromadendrene (0.78%), γ-cadinene (0.68%), 3-phenyl propionaldehyde (0.65%), chavicol (0.59%), *trans*-calamenene (0.57%), α-muurolene (0.38%), α-terpineol (0.37%), eugenol (0.29%), α-calacorene (0.28%), nerolidol (0.27%), γ-muurolene (0.26%), *epi*-cubenol (0.2%), α-humulene (0.17%), α-pinene (0.15%), (+)-aromadendrene (0.13%), camphene (0.13%), linalool (0.13%), and limonene (0.11%)	[1]
Linalool type		
Leaves (4.69%)	Cinnamon F—linalool (90.61%), citral (1.93%), coumarin (1.09%), β-caryophyllene (1.02%), anethole (1.02%), salicylaldehyde (0.77%), τ-cadinol (0.63%), linalool oxide (0.59%), α-terpineol (0.55%), isoledene (0.50%), and caryophyllene oxide (0.49%)	[15]
Leaves (4.7%)	Cinnamon 4—linalool (90.6%), citral (1.9%), coumarin (1.1%), β-caryophyllene (1.0%), anethole (1.0%), salicylaldehyde (0.8%), α-terpineol (0.6%), τ-cadinol (0.6%), caryophyllene oxide (0.5%), and isoledene (0.5%)	[11]
Leaves (2.9%)	Cinnamon CO6—linalool (86.6%), coumarin (4.1%), *trans*-cinnamaldehyde (3.1%), β-caryophyllene (1.3%), anethole (0.9%), τ-cadinol (0.7%), δ-cadinene (0.7%), salicylaldehyde (0.6%), isoledene (0.5%), linalool oxide (0.5%), and α-terpineol (0.4%)	[16]
Leaves (4.69%)	Cinnamon D—linalool (95.41%), *trans*-cinnamaldehyde (1.40%), cinnamyl acetate (1.15%), 4-allylanisole (0.61%), and α-terpineol (0.38%)	[14]
Leaves (4.69%)	Cinnamon COE—linalool (95.13%), *trans*-cinnamaldehyde, 4-allylanisole (0.56%), coumarin (0.48%), α-terpineol (0.43%), *cis*-linalool oxide (0.41%), (+)-limonene (0.40%), and τ-cadinol (0.34%)	[18]
Leaves (no information)	Cinnamon COG—linalool (96.28%), β-caryophyllene (0.48%), *trans*-cinnamaldehyde (0.38%), limonene (0.33%), 4-allylanisole (0.25%), coumarin (0.25%), α-terpineol (0.2%), α-pinene (0.24%), β-myrcene (0.14%), *cis*-linalool oxide (0.18%), β-pinene (0.12%), and δ-cadinene (0.12%)	[1]
Leaves (3.7%)	Cinnamon A—S-(+)-linalool (89.91%), *trans*-cinnamyl acetate (2.05%), *trans*-cinnamaldehyde (0.91%), *trans*-linalool oxide (0.57%), *trans*-β-caryophyllene (0.54%), α-pinene (0.50%), coumarin (0.47%), *cis*-linalool oxide (0.34%), α-terpineol (0.33%), spathulenol (0.30%), 4-allylanisole (0.24%), cubebol (0.22%), β-pinene (0.21%), α-cadinol (0.15%), *trans*-β-ocimene (0.14%), valencene (0.14%), α-humulene (0.13%), τ-cadinol (0.13%), and caryophyllene oxide (0.11%)	[28]
Leaves (3.7%)	Cinnamon B—S-(+)-linalool (87.61%), *trans*-cinnamaldehyde (1.0%), *trans*-linalool oxide (0.60%), coumarin (0.45%), α-terpineol (0.40%), *cis*-linalool oxide (0.39%), *trans*-β-caryophyllene (0.36%), α-pinene (0.37%), spathulenol (0.28%), 4-allylanisole (0.22%), β-pinene (0.16%), valencene (0.16%), α-cadinol (0.16%), and cubebol (0.13%)	[28]
Leaves (3.7%)	Cinnamon C—S-(+)-linalool (91.06%), *trans*-cinnamyl acetate (3.02%), *trans*-cinnamaldehyde (1.13%), *trans*-cinnamyl acetate (0.85%), *trans*-β-caryophyllene (0.71%), *trans*-linalool oxide (0.54%), coumarin (0.47%), α-terpineol (0.38%) α-pinene (0.37%), spathulenol (0.32%), *cis*-linalool oxide (0.31%), 4-allylanisole (0.26%), cubebol (0.24%), β-pinene (0.18%), α-cadinol (0.18%), valencene (0.17%), τ-cadinol (0.16%), α-humulene (0.16%), *trans*-β-ocimene (0.14%), and caryophyllene oxide (0.12%)	[28]
Leaves (3.7%)	Cinnamon D—S-(+)-linalool (93.42%), *trans*-cinnamaldehyde (1.08%), *trans*-cinnamyl acetate (0.63%), coumarin (0.54%), *trans*-β-caryophyllene (0.47%), α-pinene (0.36%), spathulenol (0.24%), α-terpineol (0.23%), 4-allylanisole (0.22%), cubebol (0.16%), β-pinene (0.15%), *trans*-β-ocimene (0.15%), α-cadinol (0.14%), and α-humulene (0.10%)	[28]
Leaves (3.7%)	Cinnamon E—S-(+)-linalool (88.13%), *trans*-cinnamyl acetate (2.52%), *trans*-cinnamaldehyde (0.80%), *trans*-β-caryophyllene (0.71%), α-pinene (0.57%), coumarin (0.50%), α-terpineol (0.38%), spathulenol (0.32%), *cis*-linalool oxide (0.27%), cubebol (0.27%), β-pinene (0.25%), 4-allylanisole (0.24%), *trans*-linalool oxide (0.20%), α-humulene (0.16%), valencene (0.15%), τ-cadinol (0.14%), *trans*-β-ocimene (0.13%)	[28]
Leaves (3.7%)	Cinnamon F—S-(+)-linalool (93.96%), *trans*-cinnamaldehyde (1.17%), *trans*-linalool oxide (0.52%), *trans*-β-caryophyllene (0.51%), coumarin (0.49%), α-pinene (0.44%), *cis*-linalool oxide (0.43%), *trans*-cinnamyl acetate (0.35%), α-terpineol (0.23%), 4-allylanisole (0.23%), *trans*-β-ocimene (0.22%), spathulenol (0.20%), β-pinene (0.16%), cubebol (0.13%), valencene (0.11%), α-humulene (0.10%), and α-cadinol (0.10%)	[28]
Leaves (3.7%)	Cinnamon G—S-(+)-linalool (94.11%), *trans*-cinnamaldehyde (1.57%), coumarin (0.71%), *trans*-linalool oxide (0.50%), *trans*-β-caryophyllene (0.48%), α-pinene (0.29%), *cis*-linalool oxide (0.27%), spathulenol (0.26%), 4-allylanisole (0.22%), *trans*-β-ocimene (0.16%), α-terpineol (0.16%), and cubebol (0.16%), *trans*-cinnamyl acetate (0.13%), valencene (0.12%), β-pinene (0.12%), and α-humulene (0.10%),	[28]
Leaves (3.7%)	Cinnamon H—S-(+)-linalool (93.88%), *trans*-cinnamaldehyde (1.21%), *trans*-β-caryophyllene (0.48%), *cis*-linalool oxide (0.47%), coumarin (0.45%), *trans*-cinnamyl acetate (0.45%), α-pinene (0.41%), spathulenol (0.26%), *cis*-linalool oxide (0.25%), 4-allylanisole (0.21%), α-terpineol (0.20%), *trans*-β-ocimene (0.19%), β-pinene (0.16%), cubebol (0.16%), valencene (0.12%), α-humulene (0.11%), and α-cadinol (0.10%)	[28]
Leaves (3.7%)	Cinnamon I—S-(+)-linalool (92.71%), *trans*-cinnamaldehyde (0.94%), *trans*-cinnamyl acetate (0.82%), *trans*-linalool oxide (0.54%), coumarin (0.52%), *trans*-β-caryophyllene (0.49%), α-pinene (0.29%), α-terpineol (0.28%), *cis*-linalool oxide (0.26%), spathulenol (0.26%), 4-allylanisole (0.23%), cubebol (0.21%), β-pinene (0.15%), valencene (0.13%), linalyl acetate (0.11%), *trans*-β-ocimene (0.14%), caryophyllene oxide (0.11%), α-cadinol (0.11%), and α-humulene (0.10%)	[28]
Leaves (3.7%)	Cinnamon J—S-(+)-linalool (94.18%), *trans*-cinnamaldehyde (1.19%), coumarin (0.67%), *trans*-β-caryophyllene (0.51%), *trans*-linalool oxide (0.49%), *trans*-cinnamyl acetate (0.30%), α-pinene (0.25%), spathulenol (0.24%), *cis*-linalool oxide (0.22%), 4-allylanisole (0.22%), α-terpineol (0.18%), *trans*-β-ocimene (0.16%), cubebol (0.16%), β-pinene (0.12%), valencene (0.12%), and α-cadinol (0.11%), and α-humulene (0.10%)	[28]
Leaves (3.7%)	Cinnamon K—S-(+)-linalool (91.14%), *trans*-cinnamaldehyde (1.43%), *trans*-cinnamyl acetate (1.27%), *trans*-β-caryophyllene (0.67%), coumarin (0.57%), *trans*-linalool oxide (0.55%), caryophyllene oxide (0.35%), *cis*-linalool oxide (0.31%), α-pinene (0.31%), 4-allylanisole (0.23%), α-terpineol (0.25%), spathulenol (0.22%), cubebol (0.15%), τ-cadinol (0.15%), β-pinene (0.14%), α-cadinol (0.15%), valencene (0.14%), and *trans*-β-ocimene (0.13%)	[28]
Leaves (3.7%)	Cinnamon L—S-(+)-linalool (88.56%), *trans*-cinnamyl acetate (2.40%), *trans*-cinnamaldehyde (1.01%), *trans*-β-caryophyllene (0.68%), *trans*-linalool oxide (0.50%), coumarin (0.%), α-terpineol (0.33%), *cis*-linalool oxide (0.26%), 4-allylanisole (0.26%), α-pinene (0.24%), spathulenol (0.24%), cubebol (0.21%), valencene (0.17%), *trans*-β-ocimene (0.14%), α-humulene (0.15%), β-pinene (0.15%), α-cadinol (0.13%), τ-cadinol (0.12%), and caryophyllene oxide (0.10%)	[28]
Leaves (no information)	Linalool (40.24%), *trans*-cinnamyl acetate (11.71%), camphor (9.38%), *cis*-cinnamaldehyde (6.87%), 3-phenyl-2-propenal (4.06%), caryophyllene (2.65%), coumarin (2.13%), bornyl acetate (1.72%), limonene (1.53%), α-(+)-pinene (1.38%), estragole (1.31%), caryophyllene oxide (1.0%), δ-cadinene (0.96%), copaene (0.85%), rimuen (0.78%), hexadecanoic acid (0.69%), cymene (0.68%), 1,8-cineole (0.64%), camphene (0.63%), eugenol (0.55%), acetyl eugenol (0.51%), (+)-nerolidol (0.48%), β-pinene (0.45%), geraniol acetate (0.43%), α-humulene (0.37%), γ-muurolene (0.36%), β-myrcene (0.35%), β-patchoulene (0.27%), chavicol (0.25%), aromadendrene (0.26%), *cis*-cinnamic acid (0.23%), *cis,trans*-α-farnesene (0.20%), and *trans*-cinnamaldehyde (0.17%)	[29]
Leaves (no information)	Linalool (40.24%), *trans*-cinnamyl acetate (11.71%), camphor (9.38%), cinnamaldehyde (6.87%), 3-phenyl-2-propenal (4.06%), caryophyllene (2.65%), coumarin (2.13%), bornyl acetate (1.72%), limonene (1.53%), α-(+)-pinene (1.38%), estragole (1.31%), and caryophyllene oxide (1.0%)	[30]
Camphor type		
Leaves (0.82%)	Cinnamon E—camphor (43.99%), bornyl acetate (20.81%), limonene (8.70%), terpinene-4-ol (4.22%), eugenol (2.32%), β-pinene (2.24%), α-terpineol (2.18%), salicylaldehyde (1.79%), β-caryophyllene (1.60%), τ-cadinol (1.56%), α-fenchene (1.53%), geraniol (1.39%), α-cadinol (0.97%), *trans*-cinnamaldehyde (0.96%), borneol (0.92%), γ-elemene (0.85%), α-terpinene (0.81%), caryophyllene oxide (0.75%), cinnamyl acetate (0.74%), camphene (0.63%), β-cadinene (0.56%), and guaiol (0.46%)	[15]
Leaves (0.8%)	Cinnamon 5—camphor (44.0%), bornyl acetate (20.8%), *p*-cymene (8.7%), terpinene-4-ol (4.2%), eugenol (2.3%), α-terpineol (2.2%), β-pinene (2.2%), salicylaldehyde (1.8%), τ-cadinol (1.6%), β-caryophyllene (1.6%), α-fenchene (1.5%), geraniol (1.4%) α-cadinol (1.0%), *cis*-cinnamaldehyde (1.0%), caryophyllene oxide (0.8%), cinnamyl acetate (0.7%), β-cadinene (0.6%), and camphene (0.6%)	[11]
Leaves (0.8%)	Cinnamon CO5—camphor (43.8%), bornyl acetate (20.6%), limonene (8.7%), terpinene-4-ol (4.3%), β-pinene (2.2%), α-terpineol (2.0%), geraniol (1.7%), borneol (0.9%), α-terpinene (0.8%), and camphene (0.6%)	[16]
Leaves (0.82%)	Cinnamon C—camphor (53.74%), L-bornyl acetate (22.09%), (+)-limonene (7.31%), α-terpineol (2.43%), eugenol (2.3%), salicylaldehyde (1.8%), β-caryophyllene (1.6%), τ-cadinol (1.6%), α-fenchene (1.5%), α-cadinol (1.0%), *trans*-cinnamaldehyde (1.0%), geranyl acetate (0.93%), γ-elemene (0.9%), *trans*-cinnamaldehyde (0.84%), caryophyllene oxide (0.8%), cinnamyl acetate (0.7%), β-cadinene (0.6%), cinnamyl acetate (0.58%), and guaiol (0.5%)	[14]
Leaves (0.82%)	Cinnamon COF—camphor (56.78%), L-bornyl acetate (21.53%), (+)-limonene (7.39%), terpinen-4-ol (2.01%), α-terpineol (1.89%), β-myrcene (1.42%), α-pinene (1.32%), caryophyllene oxide (1.27%), τ-cadinol (1.11%), camphene (1.01%), geranyl acetate (1.01%), geranial (0.89%), salicylaldehyde (0.85%), α-cadinol (0.77%), and borneol (0.75%)	[18]
Leaves (no information)	Cinnamon COF—D-(+)-camphor (56.63%), L-bornyl acetate (31.33%), α-terpineol (1.19%), (+)-terpinen-4-ol (1.04%), caryophyllene oxide (0.8%), coumarin (0.72%), τ-cadinol (0.64%), limonene (0.61%), α-cadinol (0.6%), cinnamyl acetate (0.59%), *p*-cymene (0.52%), α-copaene (0.49%), eugenol (0.4%), α-pinene (0.39%), L-borneol (0.35%), *trans*-cinnamaldehyde (0.35%), camphene (0.31%), δ-cadinene (0.27%), 4-allylanisole (0.24%), β-pinene (0.14%), β-caryophyllene (0.14%), nerolidol (0.12%), chavicol (0.1%), and γ-cadinene (0.1%)	[1]
Leaves (0.36%)	Cinnamon Y6—D-(+)-camphor (58.04%), L-bornyl acetate (27.90%), α-terpineol (1.08%), τ-cadinol (1.0%), caryophyllene oxide (0.92%), and coumarin (0.85%)	[2]
Mixed type		
Leaves (6.02 mL/kg in both studies)	Cinnamon A—neral (12.82%), 1,8-cineole (11.32%), linalool (9.83%), cinnamyl acetate (9.04%), cinnamaldehyde (8.35%), borneol (7.46%), geranyl acetate (6.03%), geraniol (4.73%), α-terpineol (4.62%), and benzaldehyde (0.93%)	[12,20]
Leaves (6.02 mL/kg)	Cinnamon A—neral (12.82%), 1,8-cineole (11.32%), linalool (9.83%), cinnamyl acetate (9.04%), cinnamaldehyde (8.35%), borneol (7.46%), geranyl acetate (6.03%), geraniol (4.73%), α-terpineol (4.62%), and benzaldehyde (0.93%)	[13]
Leaves (0.13%)	Cinnamon D—τ-cadinol (17.46%), α-cadinol (11.68%), bornyl acetate (9.75%), caryophyllene oxide (8.02%), *p*-allylanisole (3.80%), aromadendrene (3.03%), α-cubebene (2.95%), azunol (2.94%), isoledene (2.24%), coumarin (2.01%), γ-murrolene (1.78%), t-murrolene (1.77%), β-cadinene (1.44%), α-murrolene (1.21%), α-terpineol (0.80%), α-guaiene (0.80%), guaiol (0.68%), β-cadinol (0.61%), geraniol (0.59%), γ-elemene (0.51%), linalool (0.50%), valencene (0.47%), cyclosativene (0.38%), borneol (0.26%), *trans*-cinnamaldehyde (0.24%), and humulene (0.18%)	[15]
Leaves (0.55%)	1,8-Cineole (17.01%), spathulenol (15.7%), santolina triene (14.2%), caryophyllene oxide (11.2%), citral (6.14%), *trans*-verbenol (4.20%), α-terpineol (3.61%), *cis*-linalool oxide (3.06%), germacrene b (1.62%), 1-butenylidene-cyclohexane (1.49%), α-campholenal (1.24%), 6-camphenol (1.12%), 1,4,4-trimethylcyclohex-2-enecarboxylic acid (1.09%), sabinene hydrate (1.0%), α-cadinol (0.87%), τ-cadinol (0.79%), δ-cadinene (0.69%), geranyl acetate (0.63%), *cis*-geraniol (0.61%), and τ-cadinene (0.61%)	[31]
Leaves (0.1%)	Cinnamon 6—τ-cadinol (17.5%), α-cadinol (11.7%), bornyl acetate (9.8%), caryophyllene oxide (8.0%), anisole (3.8%), α-cubebene (3.0%), aromadendrene (3.0%), azunol (2.9%), isoledene (2.2%), coumarin (2.0%), t-muurolene (1.8%), α-β-cadinene (1.4%), muurolene (1.2%), α-terpineol (0.8%), geraniol (0.6%), linalool (0.5%), and *cis*-cinnamaldehyde (0.2%)	[11]
Leaves (0.1%)	Cinnamon CO4—τ-cadinol (17.8%), α-cadinol (11.5%), bornyl acetate (9.8%), caryophyllene oxide (8.0%), *p*-allylanisole (3.8%), α-cubebene (3.0%), aromadendrene (3.0%), azunol (2.9%), isoledene (2.2%), coumarin (2.0%), γ-muurolene (1.8%), β-cadinene (1.4%), α-guaiene (0.8%), guaiol (0.8%), α-terpineol (0.8%), geraniol (0.6%), β-cadinol (0.6%), γ-elemene (0.5%), camphor (0.5%), cyclosativene (0.4%), borneol (0.3%), *trans*-cinnamaldehyde (0.2%), and humulene (0.2%)	[16]
Leaves (0.55%)	Cinnamon F—spathulenol (20.38%), linalool (10.73%), α-terpineol (5.26%), *trans*-cinnamaldehyde (2.82%), geranyl acetate (0.73%), and cinnamyl acetate (0.61%)	[14]
Twigs (0.08%)	L-bornyl acetate (15.89%), caryophyllene oxide (12.98%), γ-eudesmol (8.03%), β-caryophyllene (6.60%), τ-cadinol (5.49%), δ-cadinene (4.79%), *trans*-β-elemenone (4.25%), cadalene (4.19%), *trans*-cinnamaldehyde (4.07%), α-copaene (3.93%), caryophylla-4(14),8(15)-dien-5,alpha-ol (3.74%), δ-cadinol (3.51%), spathulenol (2.75%), cinnamyl acetate (2.74%), *p*-allylanisole (2.45%), α-calacorene (1.88%), α-terpineol (1.67%), curcumene (1.67%), α-caryophyllene (1.43%), guaiol acetate (1.37%), *e*-nerolidol (1.05%), elemicin (0.85%), l-borneol (1.34%), and eugenol (0.95%)	[4]
Leaves (1.03%)	Cinnamon COA—geranial (24.13%), neral (13.09%), spathulenol (12.55%), 1,8-cineole (9.21%), linalool (5.16%), geranyl acetate (4.64%), L-bornyl acetate (4.13%), α-terpineol (2.78%), geraniol (2.02%), caryophyllene oxide (1.89%), benzaldehyde (1.18%), α-cadinol (1.13%), *trans*-linalool oxide (1.11%), δ-cadinene (1.09%), geranyl formate (0.98%), borneol (0.97%), *cis*-linalool oxide (0.91%), terpinen-4-ol (0.74%), and τ-cadinol (0.72%)	[18]
Leaves (no information)	Cinnamon COD—L-bornyl acetate (21.98%), α-cadinol (11.15%), τ-cadinol (10.9%), caryophyllene oxide (5.62%), aromadendrene (5.52%), α-copaene (4.34%), coumarin (3.64%), γ-muurolene (2.66%), 4-allylanisole (2.49%), *trans*-calamenene (2.23%), γ-cadinene (1.82%), α-muurolene (1.13%), α-terpineol (0.74%), cyclosativene (0.67%), β-selinene (0.59%), and α-calacorene (0.58%)	[1]
Leaves (no information)	L-bornyl acetate (16.6%), τ-cadinol (14.2%), α-cadinol (8.8%), caryophyllene oxide (5.5%), alloaromadendrene (5.0%), coumarin (4.3%), cadalene (4.1%), α-copaene (2.8%), γ-muurolene (2.7%), 4-allylanisole (1.9%), *trans*-calamenene (1.9%), γ-cadinene (1.9%), 1,10-di-*epi*-cubenol (1.2%), α-muurolene (1.1%), spathulenol (1.0%), α-terpineol (0.7%), β-selinene (0.6%), α-calacorene (0.6%), (+)-cyclosativene (0.5%), L-borneol (0.4%), β-bourbonene (0.4%), nerolidol (0.4%), *trans*-cinnamyl acetate (0.3%), (+)-aromadendrene (0.3%), *cis*-verbenol (0.2%), D-(+)-camphor (0.2%), and benzyl benzoate (0.2%)	[32]

* Information about volatile compounds was added for relative content > 0.09%. The letters A, B, C, ……, in cinnamon species refer to clones of cinnamon investigated in each study and were defined by authors in each study.

**Table 2:** The reported non-essential oil metabolites of *C. osmophloeum,* the plant material, type of extract, and methods of characterization/identification.

Plant Material	Type of Extract	Compound	Identification Method	Reference
Leaves	Methanol extract (chloroform and *n*-butanol fractions)	**1**–**4**	Spectral data (^1^H, ^13^C, COSY, HMBC, HMQC, and NOESY NMR; UV; and HRMS) and acid hydrolysis	[33]
Leaves	Methanol extract (*n*-butanol fraction)	**2** and **3**	Spectral data (no more information)	[34]
Leaves	Water extract	**1** and **2**	HPLC-HRMS	[35]
Twigs	Ethanol extract (*n*-butanol fraction)	**5**	Spectral data (^1^H, ^13^C, HMBC, and HSQC NMR; UV; and HRMS)	[36]
Leaves	Water extract (ethyl acetate fraction)	**1** and **5**	Spectral data (^1^H, ^13^C, HSQC and HMBC NMR, and HRMS)	[37]
Twigs	Ethanol extract (*n*-butanol fraction)	**1**–**3** and **5**–**10**	Spectral data (^1^H, ^13^C, NOESY, COSY, HSQC, and HMBC NMR; and HRMS)	[38]
Leaves	Hydrosol	**11**–**25**	UPLC-MS/MS	[39]
Leaves	Hydrosol	**11**–**24**	UPLC-MS/MS	[40]
Bark and twigs	Acetone extract (*n*-butanol fraction)	**26**–**27**	Spectral data (^1^H and ^13^C NMR)	[41]
Bark and roots	Ethanol extract (chloroform and *n*-butanol fractions)	**28**–**39**	Spectral data (^1^H and ^13^C NMR, UV, IR, and HRMS)	[42]
Stems	Methanol extract (chloroform fraction)	**40**	Spectral data (^1^H and ^13^C NMR, UV, IR, and HRMS)	[43]

Abbreviations: COSY, Correlated Spectroscopy; HMBC, Heteronuclear Multiple Bond Correlation; HMQC, Heteronuclear Multiple Quantic Coherence; NOESY, Nuclear Overhauser Effect Spectroscopy; NMR, Nuclear Magnetic Resonance; UV, Ultraviolet, HRMS, High-Resolution Mass Spectrometry; HSQC, Heteronuclear Single Quantum Coherence; IR, Infrared; HPLC, High-Performance Liquid Chromatography; UPLC, Ultra Performance Liquid Chromatography.

**Table 3:** Biological activities of extracts and essential oils from *C. osmophloeum* and their constituents.

Material	Study Model	Dose and/or Concentration	Effects	Reference
Hexane, ethyl acetate, and methanol extracts from the bark	LPS/IFN-γ-activated murine peritoneal macrophages	2.5–12.5 μg/mL or 25–155 μg/mL	Inhibited NO production via suppression of iNOS. Reduced TNF-α and IL-12 levels	[44]
Water extracts from the leaves	In vitro antioxidant activities	12.5–100 μg/mL	Demonstrated beneficial antioxidant activity, DPPH free radical scavenging, and superoxide scavenging	[37]
Water extracts from the leaves	In vitro antioxidant activities	0.01–0.5 mg/mL	Presented high ability to scavenge DPPH radicals, metal chelation, and reducing power	[26]
Essential oils from leaves and their constituents (*trans*-cinnamaldehyde, τ-cadinol, and α-cadinol)	LPS-activated RAW 264.7 macrophages	5–100 µg/mL and 12.5–200 µM	Inhibited NO production	[1]
Essential oils from twigs and their constituents (*trans*-cinnamaldehyde, caryophyllene oxide, L-borneol, L-bornyl acetate, eugenol, β-caryophyllene, *E*-nerolidol, and cinnamyl acetate)	LPS-activated RAW 264.7 macrophages and HepG2 cells	10–25–50 µg/mL and 200–250 µg/mL	Demonstrated anti-inflammatory activities and cytotoxicity against HepG2 cells, and suppressive effect on NO production	[4]
Essential oils from leaves and their constituents (*trans*-cinnamaldehyde and D-(+)-camphor)	In vivo antioxidant activities in *C. elegans*	10–20 µg/mL and 1–20 µM	Enhanced the oxidative stress resistance of *C. elegans* and induced expressions of SOD and GST	[2]
Essential oil from leaves	LPS-stimulated J774A.1 cells	5–60 µg/mL	Inhibited proIL-1 expression and levels of TNF-α, IL-1β, and IL-6	[31]
Essential oil from leaves	Endotoxin-induced intestinal injury in mice	6.5–26 mg/kg	Reduced levels of TNF-α, IL-1β, IL-18, IFN-γ, and NO. Inhibited the expression of TLR4, MyD88, MD2, ASC, caspase-1, NOD, and NLRP3. Inhibited the activation of NF-κB and caspase-1	[30]
Essential oil and cinnamaldehyde from leaves	STZ-induced diabetic rats	12.5–50 mg/kg	Reduced blood glucose and fructosamine. Elevated plasma insulin levels. Reduced levels of TBARS, IL-1β, and NO. Increased activities of SOD, GPx, GRd, and total GSH	[29]
Cinnamaldehyde from leaves	DSS-induced colitis in mice	100 mg/kg	Alleviated colitis, downregulated the activation of NLRP3 inflammasome, and enhanced the autophagic response	[67]
Cinnamaldehyde from leaves	*S. sonnei*-infected macrophages	10–40 µM	Inhibited the NLRP3 inflammasome, caspase-1, and reduced IL-1β, IL-6, TNF-α, and IL-18 expression. Inhibited pyroptosis; decreased caspase-11 and Gasdermin D activation. Reduced lysosomal damage, enhanced autophagy, and increased phagocytosis and bactericidal activity of macrophages	[69]
Cinnamaldehyde and linalool from leaves	Endotoxin-induced mice	0.45–0.9 mg/kg or 2.6–5.2 mg/kg	Reduced levels of NO, IL-1β, IL-18, TNF-α, IFN-γ, and HMGB-1. Inhibited expression of TLR4, MyD88, MD2, NOD, NLRP3, ASC, and caspase-1. Suppressed NF-κB activation and caspase-1 activity	[7]
Cinnamaldehyde from leaves	LPS- or LTA-stimulated J774A.1 cells and LPS-stimulated human blood monocytes	8–80 µM	Inhibited secretion of IL-1β, TNF-α, and IL-6. Reduced proIL-1 expression, MAPK phosphorylation, and ROS release	[74]
Flavonol glycosides from leaves	LPS/IFN-γ-activated murine macrophages	2.5–100 µM	Inhibited production of NO, TNF-α, and IL-12	[33]
Cinnamaldehyde from leaves	AGE-treated HK-2 cells	0.1–100 µM	Reversed the inhibition of NO generation; induced cGMP synthesis and PKG activation. Reversed JAK2-STAT1/STAT3 activation, RAGE/p27^Kip1^/collagen IV levels, and cellular hypertrophy. Suppressed STAT activation and increased SOCS-3	[87]
Constituents of essential oil from leaves (aromadendrene, τ-cadinol, α-cadinol and *trans*-cinnamaldehyde)	LPS/D-GalN-induced acute hepatitis in mice	10–200 µmol/kg	Decreased AST, ALT, TNF-α, and IL-6 levels. Reduced the expressions of cleaved caspase-3 and PARP	[89]

Abbreviations: ALT, alanine aminotransferase; ASC, apoptosis-associated speck-like protein containing a caspase recruitment domain; AST, aspartate aminotransferase; cGMP, cyclic guanosine monophosphate; D-GalN, D-galactosamine; DPPH, 2,2-diphenyl-1-picrylhydrazyl; DSS, dextran sulfate sodium; GPx, glutathione peroxidase; GRd, reduced glutathione; GSH, glutathione; GST, glutathione S transferase; HMGB-1, high mobility group box-1 protein; IFN-γ, interferon-gamma; IL, interleukin; JAK2, Janus kinase 2; LPS; lipopolysaccharide; LTA, lipoteichoic acid; MAPKs, mitogen-activated protein kinases; MD2, myeloid differentiation factor 2; MyD88, myeloid differentiation primary response 88; NF-κB, nuclear factor kappa B; NLRP3, NOD-like receptor protein 3; NO; nitric oxide; iNOS; inducible nitric oxide synthase; PARP, poly-ADP ribose polymerase; PKG, protein kinase G; RAGE, advanced glycation end products receptor; ROS, reactive oxygen species; SOCS-3, suppressor of cytokine signaling 3; SOD, superoxide dismutase; STAT1/3, signal transducer and activator of transcription 1/3; STZ, streptozotocin; TBARS, thiobarbituric acid reactive species; TLR4, toll-like receptor 4; TNF-α, tumor necrosis factor-alpha.
